# Contrasting biological potency of particulate matter collected at sites impacted by distinct industrial sources

**DOI:** 10.1186/s12989-016-0176-y

**Published:** 2016-12-01

**Authors:** Errol M. Thomson, Dalibor Breznan, Subramanian Karthikeyan, Christine MacKinnon-Roy, Ngoc Q. Vuong, Ewa Dabek-Zlotorzynska, Valbona Celo, Jean-Pierre Charland, Prem Kumarathasan, Jeffrey R. Brook, Renaud Vincent

**Affiliations:** 1 Environmental Health Science and Research Bureau, Health Canada, Ottawa, Ontario K1A 0K9 Canada; 2 Analysis and Air Quality Section, Air Quality Research Division, Atmospheric Science and Technology Directorate, Environment and Climate Change Canada, Ottawa, ON K1A 0H3 Canada; 3 Air Quality Processes Research Section, Air Quality Research Division, Atmospheric Science and Technology Directorate, Environment and Climate Change Canada, Toronto, ON M3H 5T4 Canada

**Keywords:** Air pollution, Particle, Size, Source, Industry, Toxicity, Inflammation, Metals, Polycyclic aromatic hydrocarbons, Endotoxin

## Abstract

**Background:**

Industrial sources contribute a significant proportion of anthropogenic particulate matter (PM) emissions, producing particles of varying composition that may differentially impact health. This study investigated the in vitro toxicity of ambient PM collected near industrial sites in relation to particle size and composition.

**Methods:**

Size-fractionated particles (ultrafine, PM_0.1–2.5_, PM_2.5–10_, PM_>10_) were collected in the vicinity of steel, copper, aluminium, and petrochemical industrial sites. Human lung epithelial-like A549 and murine macrophage-like J774A.1 cells were exposed for 24 h to particle suspensions (0, 30, 100, 300 μg/cm^2^). Particle potency was assessed using cytotoxic (resazurin reduction, lactate dehydrogenase (LDH) release) and inflammatory (cytokine release) assays, and regressed against composition (metals, polycyclic aromatic hydrocarbons (PAHs), endotoxin).

**Results:**

Coarse (PM_2.5–10_, PM_>10_) particle fractions were composed primarily of iron and aluminium; in contrast, ultrafine and fine (PM_0.1–2.5_) fractions displayed considerable variability in metal composition (especially water-soluble metals) across collection sites consistent with source contributions. Semi-volatile and PM-associated PAHs were enriched in the fine and coarse fractions collected near metal industry. Cell responses to exposure at equivalent mass concentrations displayed striking differences among sites (*SITE x SIZE* and *SITE x DOSE* interactions, *p* < 0.05), suggesting that particle composition, in addition to size, impacted particle toxicity. While both J774A.1 and A549 cells exhibited clear particle size-dependent effects, site-dependent differences were more pronounced in J774A.1 cells, suggesting greater sensitivity to particle composition. Plotting particle potency according to cytotoxic and inflammatory response grouped particles by size and site, and showed that particles of similar composition tended to cluster together. Cytotoxic effects in J774A.1 cells correlated with metal and PAH content, while inflammatory responses were associated primarily with endotoxin content in coarse particles.

**Conclusions:**

Industrial sources produce particulate emissions with varying chemical composition that differ in their in vitro potency in relation to particle size and the levels of specific constituents.

**Electronic supplementary material:**

The online version of this article (doi:10.1186/s12989-016-0176-y) contains supplementary material, which is available to authorized users.

## Background

On the basis of associations between particulate matter levels and morbidity and mortality [1–4], air quality standards in North America and Europe are expressed as mass concentrations for a given size range (i.e., PM_10_ and PM_2.5_; particles with an aerodynamic diameter less than 10 μm and 2.5 μm respectively). It is clear, however, that biological responses to particulate matter are dependent not only on size, but also on other physicochemical characteristics such as inorganic and organic composition, which are impacted by mode of generation, and therefore source. Industrial sources are major contributors of particulate matter pollution, and in Canada account for an estimated 64% of total emitted particulate matter and 39% of PM_10_ (Environment Canada (2012)). An understanding of the relative contribution of sources and specific particle constituents to the toxic potency of airborne particulate matter could complement epidemiological studies and exposure data to ultimately inform regulatory initiatives aimed at targeting specific emission sources [5].

Particulate matter is a heterogeneous mixture composed of carbonaceous combustion materials such as polycyclic aromatic hydrocarbons, biogenic material, salts, and other inorganic materials such as transition metals. Particle composition varies geographically and temporally both in chemical composition and size distribution, and these differences are associated with variance in the magnitude of health impacts observed in epidemiological studies [3, 6, 7]. Differences in effects elicited by sub-components of the same particle mix may reflect particle size (e.g., the increased surface area to mass ratio of smaller particles resulting in more surface activity or adsorbed substances; differential particle deposition in the respiratory tract), particle number, composition, or combinations of these factors. We and others have shown previously that the potency of respirable fine particles from different geographical locations is related to their physicochemical characteristics, in particular to the profiles of bioavailable transition metals and polycyclic aromatic hydrocarbons (e.g., [8–12]. While soluble metals appear to play a key role in both acute lung injury [13, 14] and cardiovascular effects [15, 16] of particle exposure in rodents, some studies have found that insoluble particle components are more closely associated with cytotoxic and inflammatory responses in vivo and in vitro [17, 18]. Microbial components (e.g., endotoxin) associated with particles have also been shown to contribute to biological effects of ambient particles, in particular to their inflammogenic potential [9, 19]. Responses of cells that have direct contact with inhaled particles, such as alveolar macrophages and lung epithelial cells, may therefore depend on various particle properties (e.g., size, composition) and their interaction with cell-type-specific characteristics.

Several studies suggest that particulate emissions from specific industrial sources have significant population health impacts. Closure of a steel mill in Utah was associated with a reduction in respiratory and cardiovascular mortality, with daily mortality associated with PM_10_ levels [20]. Comparison of the biological potency of aqueous extracts of particulate samples collected on filters before, during, and after closure and resumption of mill activity revealed increased toxicity (according to lactate dehydrogenase, interleukin (IL)-6 and IL-8 release) associated with higher levels of lead, copper, zinc, and iron (each 2-3-fold higher) when the mill was operating [21]. A study examining health effects of living within pollution gradients of steel industry in Hamilton, Ontario, found increased relative risk of premature mortality associated with higher levels of total suspended particulate matter [22]. Proximity to copper refineries was associated with higher levels of lead, copper, arsenic, and zinc in ambient particles, and increased respiratory mortality [23], and closure of a copper refinery was associated with reduced respiratory and cardiovascular mortality [24]. This was attributed to reduced sulfate levels, although it was noted that transition metals (for which data were not available) would also have been expected to decrease during this period [25]. Changes in heart rate and heart rate variability observed in mice exposed to concentrated fine particulate matter in Tuxedo, New York, were associated with nickel, with 72 h back trajectories suggesting that the air mass passed over the nickel smelter in Sudbury, Ontario [26]. These studies implicate industrial emissions in the health impacts associated with exposure to particulate air pollution. However, to our knowledge there has been no direct comparison of the composition and relative toxicity of size-fractionated particles collected in the vicinity of multiple distinct industrial point sources. Such a study could provide valuable information on the links between sources, physicochemical characteristics of the particles, and toxic potency that underlie associations between exposure to particulate matter and adverse health effects.

Ambient particulate matter at any given site would be expected to be impacted by fixed regional sources, mobile transportation sources, and long-range transit of particles, in addition to local point sources. Nevertheless, airborne particulate matter collected downwind of specific industrial sources for an extended period of time should be enriched for particles generated by that source. In vitro models provide an effective platform for screening and comparing biological responses to materials of differing composition. Using such models, we have previously shown that particles collected at industrial, high traffic, and residential sites within a small urban area can vary significantly in their composition and potency [12]. In the present study our objective was to compare the relative toxic potency of size-fractionated ambient particles collected at sites impacted by distinct industrial emission sources and investigate determinants of toxicity. We hypothesised that particles collected in the vicinity of different industrial sources would differ in their composition and in their toxic potency in biological systems. By sampling the air at sites in close proximity to specific industrial sources, our intention was to collect size-fractionated samples with distinct chemical composition that reflected a strong signal from the industrial site in question. A panel of cytotoxic and inflammatory assays was used to generate a response signature for each particulate sample, enabling regression of biological endpoints against particle constituents to investigate potency drivers. Our results show that particles collected in the vicinity of industrial sites display clear size- and site-dependent differences in composition and biological potency.

## Methods

### Particle collection and extraction

A ChemVol High Volume Cascade Impactor [27] was used to collect size-fractionated particles at ground level in the vicinity of five industrial sites. These were: a steel mill in Hamilton (Hamilton Beach, HB), Ontario (5 samples between October 24 and December 6, 2007); a petrochemical refinery in Sarnia (SR), Ontario (7 samples between January 25 and March 11, 2008), and another in Montréal (MA), Québec (5 samples between May 4 and June 8, 2009); a copper smelter in Montréal (MC), Québec (5 samples between February 24 and March 30, 2009); and an aluminum refinery in Shawinigan (SW), Québec (5 samples between June 18 and July 23, 2009). The sampler employed three impaction stages with polyurethane foam collection surfaces with target cut points at 10, 2.5, and 0.1 μm mass median aerodynamic diameter followed by collection of ultrafine particles on polypropylene filters, producing particle fractions with aerodynamic diameter >10 μm (PM_>10_ or super-coarse), between 2.5 and 10 μm (PM_2.5–10_ or coarse), between 0.1 and 2.5 μm (PM_0.1–2.5_ or fine), and <0.1 μm (ultrafine particles, UFP). Collection efficiency curves for the ChemVol impaction stages were reported previously by Demokritou et al. (2002). A high capacity Roots™ Universal RAI Rotary Positive Blower was used to maintain the highest flow rate possible through the ChemVol impaction slits. The main restriction and greatest pressure drop was provided by the 0.1 μm cut stage, which limited the flow rate to the design flow of about 900 L/min according to the manufacturer (Rupprecht and Patashnick, Albany, NY). Samples were collected over a period of five weeks at each site, with the exception of Sarnia where the collection period was for seven weeks, with collection substrates replaced each week. Field blank filters were transported to each site, but remained unexposed to ambient air. They underwent all other steps followed by the samples (i.e., cleaning, handling in the laboratory, shipping, storage at the field site and in the laboratory, and extraction). All collection surfaces were precleaned prior to sampling with repeated sonication in Milli-Q-grade deionized, sterile water (18.2 MOhm.cm resistivity), and Omnisolv HR-GC-grade methanol (EMD Millipore, Etobicoke, ON, Canada), followed by drying under nitrogen and final evaporation of solvent in drying oven. Cleaned substrates were stored in sterile sample bags wrapped in foil to protect from photodegradation.

Particles were recovered from polyurethane foam and filters by aqueous extraction and sonication as previously described [12]. Samples collected across weeks at each site were extracted together, such that the extracted particles represent the full collection period. Extracted particles were vacuum-dried (22 h, 37 °C) using MiVac SpeedTrap™ vacuum concentrator (Genevac Ltd., Ipswitch, Suffolk, UK). Extraction efficiencies of particle mass determined by comparing filter masses pre- and post-extraction were 96 ± 19% from polyurethane foam and 37 ± 13% polypropylene filters. Ultrasonic agitation and mechanical manipulation of the substrates during particle extraction resulted in generation of a small amount of debris that would contribute to the extracted particle mass. Extraction of field blank (unexposed) polyurethane foam and polypropylene filters indicated that such filter material contributed 1–8% of extracted mass. Debris were removed by filtration of the polyurethane foam extracts using a sterile 40 μm nylon filter (BD Falcon Cell Strainer, Corning Life Sciences, Corning, NY), while polypropylene filter extracts were filtered sequentially through 40 μm and 5 μm filters (Target Syringe Filter, National Scientific Co., Rockwood, TN). Average particle yields (final particle mass recovered relative to initial mass extracted) from polyurethane foam and polypropylene filters were 90 ± 7% and 86 ± 9% respectively. Standard reference materials (SRM) TiO_2_ (SRM-154b) and SiO_2_ (SRM-1879a) were obtained from the National Institute of Standards and Technology (NIST, Gaithersburg, MD). Preparation and characterisation of the urban particulate matter standard EHC-6802 was described previously [28].

### Characterisation of particle composition

All analyses of particle composition were performed on particles extracted as described above. For metals analyses, samples were first ultrasonically extracted with 2 mL of double deionized water (DDW, resistivity > 18 MOhm cm) for 30 min at room temperature. Both solid material and aqueous extract were quantitatively transferred to a centrifuge tube followed by dilution to 9 mL using DDW, and centrifuged for 5 min at 5000 rpm. An aliquot of supernatant (8 mL) was taken and acidified with 1% (v/v) HNO_3_ [29, 30] for further analysis of water-soluble metals using ICP-MS. The remainder of the sample (solid residue and aqueous extract) was quantitatively transferred into a digestion vial and evaporated to almost dryness. Samples were then digested and analyzed by ICP-MS as previously described [12]. The obtained results were corrected for the water-soluble metals present in the analyzed samples (1 mL aqueous extract) to calculate the concentration of non-water-soluble metals. All measurements were performed using a 7500ce ICP–MS system (Agilent Technologies, Wilmington, DE, USA). The octopole collision/reaction system (ORS), was pressurized with He gas for analysis of V, As and Cr, and with H_2_ for analysis of Fe and Se. Internal standardization with 0.5 mg/L solution of ^45^Sc, ^89^Y, ^115^In, and ^165^Ho was used to correct for instrument drift and nonspectral interferences. Quality control samples were used to determine the accuracy and precision of chemical analysis, and to diagnose contamination.

For polycyclic aromatic hydrocarbons (PAHs), particle samples were spiked with 50 μL of isotopically labeled PAH surrogate standards for recovery correction. PAH analyses were performed on PM_0.1–2.5,_ PM_2.5–10_ and PM_>10_ fractions but not ultrafine (PM_<0.1_) particles due to lack of material for this size fraction. Samples were extracted by Soxhlet apparatus in 350 mL of cyclohexane for 16 to 20 h. Following evaporation to ~5 mL, the extract was subjected to activated silica gel column chromatography for fractionation of the desired target analytes using a suite of solvents of increasing polarity as previously described [12]. The purified extract, to which 50 μL d10-fluoranthene (10 ng/μL) was added, was analysed for PAHs by GC/MS using low resolution Agilent 7890A GC interfaced directly to Agilent 5975C Mass Selective Detector under conditions previously described [12]. Quality control samples were used to determine the accuracy and precision of the chemical analyses. Field blank filter samples were used to assess and correct for background concentrations.

Endotoxin levels were assessed using the Limulus Amebocyte Lysate (LAL) chromogenic quantitation kit (Lonza, Walkersville, MD, USA) and quantified with a Synergy 2 multi-mode plate reader (Bio-Tek, Winooski, VT, USA) as described previously [12].

### In vitro exposures

Human lung epithelial-like (A549; ATCC, CCL-185) and murine macrophage-like (J774A.1; ATCC, TIB-67) cell lines (American Type Culture Collection, Manassas, VA, USA) were propagated in Dulbecco’s Modified Eagle’s Medium (Fisher Scientific) containing phenol red and 4.5 g/L glucose and supplemented with FBS (10% v/v, non-heat inactivated; Fisher Scientific) and Pen Strep (100 U/ml penicillin-G, 100 mg/ml streptomycin; Sigma-Aldrich Canada, Oakville, ON) for A549 cells and Gentamicin (50 μg/mL; Sigma-Aldrich Canada, Oakville, ON) for J774A.1 cells respectively, in 75 cm^2^ tissue culture flasks (Corning, NY, USA) at 37 °C, 5% CO2, and 95% relative humidity. Phenol red was excluded for particle exposures and bioassays to avoid interference with the detection methods. Cells were seeded in 96-well black-walled clear-bottom cell culture plates (BD Biosciences, Mississauga, ON) at 100 μL/well (A549, 2 x 10^4^ cells/well, 6 x 10^4^ cells/cm^2^; J774A.1, 4 x 10^4^ cells/well, 12 x 10^4^ cells/cm^2^) of complete medium, and incubated at 37 °C for 24 h. On the day of exposure, particle suspensions were thawed, sonicated for 20 min in an ice-cold ultrasonic water bath, diluted in complete media devoid of serum, and sonicated for a further 5 min immediately prior to cell dosing. Cell monolayers were exposed to 100 μl particle suspensions resulting in 0, 30, 100, and 300 μg/cm^2^ in a final volume of 200 μl (with a 5% final concentration of serum). Polyurethane foam and polypropylene field blank filter extracts resuspended according to the mean volume used to resuspend extracted particles for each size fraction were compared alongside particles for all toxicity experiments to test for biological effects of any residues from collection substrates. Doses were selected to range from levels that produce negligible effects to levels that produce measurable effects according to the cytotoxicity assays employed so that dose–response relationships could be evaluated [12]. Cells were incubated at 37 °C for 24 h prior to assessment of cytotoxicity and inflammatory effects, a time point selected to allow for cytotoxic and inflammatory responses to develop [12]. Three independent exposure experiments were conducted for each cell line, with duplicate technical replicates included on each plate.

### Cytotoxicity assays

Aliquots of supernatants clarified by centrifugation at 350 × g for 5 min were used in the lactate dehydrogenase (LDH) release and inflammatory cytokine assays. Metabolic activity was assessed in cells after the addition of the resazurin reagent mixture (Alamar Blue, Fisher Scientific). Reduction of the dye resazurin to resorufin was calculated by fluorescence reading at 3 h minus baseline fluorescence measured at λEx = 530–540 nm and λEm = 590–600 nm. Following removal of the supernatant, cells were washed in serum-free medium (15 min incubation at 37 °C), lysed in a buffer containing 100 mM MgCl_2_ and 0.025% Triton X-100 in PBS at room temperature for 5 min, and centrifuged for 10 min at 1700 × g. The obtained supernatants and lysates were transferred to 96-well conical bottom plates and clarified by centrifugation for 5 min at 350 × g. LDH levels were measured using the CytoTox 96 colorimetric assay (Promega Corporation, Madison, WI) as previously described [12], and LDH release was calculated as a fraction of total LDH activity recovered in supernatant and cell lysate.

### Cytokines

Levels of IL-1α, IL-1β, IL-2, IL-3, IL-4, IL-5, IL-6, IL-9, IL-10, IL-12 p70, IL-13, IL-17, eotaxin, G-CSF, GM-CSF, IFNγ, KC, MCP1, MIP-1α, MIP-1β, RANTES, and TNF were assessed in J774A.1 cell supernatants using the Bio-Plex Pro Mouse Cytokine 23-plex Assay (Bio-Rad Laboratories (Canada) Ltd., Mississauga, Ontario, Canada) on a Bio-Plex 200 multiplex luminescence assay system (Bio-Rad Laboratories (Canada) Ltd.) following the manufacturer’s protocol.

### Potency estimates

Cytotoxicity endpoints (resazurin reduction, lactate dehydrogenase release) and cytokine levels were normalized to the mean of the respective controls to generate fold-change values for each particle dose. Potency estimates (β) of the exposure-response relationship were derived from the following equation: fold-change = (Dose + 1)^β^ where β is the slope of the relationship on the logarithmic scale [8]. As higher potency is reflected by a greater negative slope in the resazurin reduction assay and a positive slope in the LDH release assay, potency estimates for resazurin reduction were multiplied by −1 prior to averaging of cytotoxic potencies across assays to generate a consensus cytotoxic potency for each particle.

### Statistical analyses

Cytotoxicity and cytokine dose–response data for the size-fractionated particle exposures were assessed for statistically significant effects by three-way ANOVA with *Dose* (0, 10, 30, 100 μg/cm^2^), *Site* (Hamilton beach (HB), Montréal petrochemical industry (MA), Montréal copper refinery (MC), Sarnia petrochemical industry (SR), Shawinigan aluminium smelter (SW)) and *Size* (UFP, PM_0.1–2.5_, PM_2.5–10_, PM_>10_) as factors. For the particle standards, two-way ANOVA was performed with *Particle* (EHC-6802, TiO_2_, SiO_2_) and *Dose* (0, 10, 30, 100 μg/cm^2^) as factors. Datasets not meeting the assumptions of normality and equal variance for ANOVA were transformed prior to analyses. Pairwise multiple comparisons were carried out using the Holm-Sidak procedure as directed by significant factor interactions or main effects in the ANOVA to elucidate the pattern of significant effects (α = 0.05). Correlations were performed between toxicological endpoints and particle chemistry using Pearson’s Product Moment Correlation and Best Subset Regression. All statistical analyses were conducted using SigmaPlot version 13 (Systat Software, Inc., San Jose, CA, USA). Clustering of particles based on composition was conducted using Minitab, version 15 (Minitab Inc., State College, PA, USA). Heatmap software was used to visualise inflammatory cytokine potency data (http://www.hiv.lanl.gov/content/sequence/HEATMAP/heatmap.html; Los Alamos National Laboratory, Los Alamos, NM, USA).

## Results

### Chemical analyses of particles from sites impacted by industrial sources

Levels of metals, PAHs, and endotoxin were analysed in each particle (summarised in Table 1).

**Table 1:** Composition of size-fractionated particles collected in the vicinity of industrial sites: total metals, endotoxin, polycyclic aromatic hydrocarbons

	Total Metals^a^ (μg/g)				Endotoxin (EU/ml/μg PM)				PAHs^b^ (μg/g of PM)		
	UFP	PM_0.1–2.5_	PM_2.5–10_	PM_>10_	UFP	PM_0.1–2.5_	PM_2.5–10_	PM_>10_	PM_0.1–2.5_	PM_2.5–10_	PM_>10_
Hamilton Beach (HB)	29512	32675	86365	72230	0.017	0.038	0.082	0.072	151	90	22
Montréal (MA)	12827	39228	59533	56430	0.019	0.047	0.074	0.080	28	74	40
Montréal (MC)	14467	19758	44853	36381	0.009	0.051	0.053	0.019	100	79	93
Sarnia (SR)	5346	9965	28264	18842	0.006	0.005	0.037	0.054	40	51	96
Shawinigan (SW)	19612	36537	87836	110990	0.010	0.044	0.046	0.078	111	413	305

^a^See Additional file 1 for complete description of metals analysed

^b^See Additional file 2 for complete description of PAHs analysed

There was considerable variability in the concentration of metals per unit mass of particulate sample across sites. Hamilton (HB) and Shawinigan (SW) had the highest total metal content across size fractions (with the exception of high levels in the fine fraction from the Montréal petrochemical site (MA)), followed by the two Montréal sites (MA, MC), and lastly Sarnia (SR), which had considerably lower metal content than all other sites. The amount of PAHs in extracted particles was also variable, with no clear trends across size fractions or sites beyond relatively high levels recovered in the coarse and super-coarse fractions in Shawinigan. Endotoxin was detected in all samples, and tended to increase with particle size (*Size* main effect, *p* < 0.001), with significantly higher levels in PM_2.5–10_ and PM_>10_ compared to the ultrafine fraction (*p* < 0.05, Holm-Sidak pairwise comparison). Detailed data on metal and PAH content are provided in Additional files 1 and 2 respectively.

**Fig. 1:**
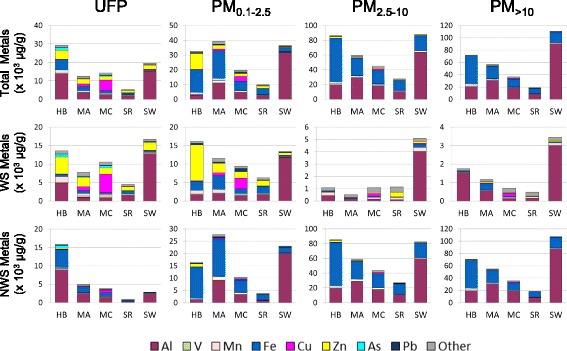
Metal composition of particles collected in the vicinity of industrial sites. The elemental composition of particulate samples according to total metals, water-soluble (WS) metals, and non-water-soluble (NWS) metals was assessed across particle size-fractions. HB, Hamilton Beach steel mill; MA, Montréal petrochemical refinery; MC, Montréal copper smelter; SR, Sarnia petrochemical refinery; SW, Shawinigan aluminum smelter

Comparison of metal content across sites revealed size-dependent contrasts in composition and solubility (Fig. 1). Compared on an equivalent mass concentration basis, PM_2.5–10_ and PM_>10_ fractions displayed total metal content 2–3 times higher than UFP and PM_0.1–2.5_ fractions. However, whereas metals in the PM_2.5–10_ and PM_>10_ fractions consisted largely of non-water-soluble iron and aluminium, metal solubility was markedly higher in UFP and PM_0.1–2.5_ fractions. The UFP and PM_0.1–2.5_ fractions were also considerably more variable in composition, with the most striking differences in composition observed in the water-soluble fractions. Notable differences included relatively high levels of zinc, manganese, and arsenic in samples collected near Hamilton steel industry; copper, arsenic, and lead in samples collected near the Montréal copper refinery and to a lesser extent in samples collected near Montréal petrochemical industry; higher cadmium and cobalt in samples collected near the Montréal petrochemical site; and the predominance of aluminum in samples collected near the Shawinigan aluminium refinery (complete metal analyses provided in Additional file 1). Clustering of particles according to the composition of water-soluble metals showed that UFP and PM_0.1–2.5_ particles were more different from each other and from all other particles than any of the PM_2.5–10_ and PM_>10_ samples (Additional file 3). Similar clustering according to size fraction was observed for total metals and non-water-soluble metals, although clusters were less defined. Analysis of covariance of water-soluble elements revealed good correlations (>85% covariance) among a number of sets of elements, including aluminum and beryllium; chromium, cobalt, molybdenum, and cadmium; vanadium, arsenic, lead, and copper; and manganese, zinc, and thallium (Additional file 4).

**Fig. 2:**
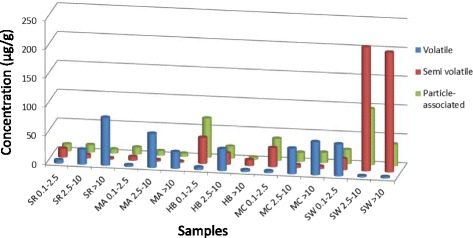
Polycyclic aromatic hydrocarbon (PAH) content of particles collected in the vicinity of industrial sites. PAHs were grouped according to volatility (volatile: acenaphthene, fluorine, phenanthrene, anthracene; semi-volatile: fluoranthene, pyrene, retene, benz(a)anthracene, chrysene; particulate-associated: benzo(b)fluoranthene, benzo(k)fluoranthene, benzo(a)pyrene, indeno(123-cd)pyrene, dibenz(a,h)anthracene, benzo(ghi)perylene) and compared across sites and size-fractions. HB, Hamilton Beach steel mill; MA, Montréal petrochemical refinery; MC, Montréal copper smelter; SR, Sarnia petrochemical refinery; SW, Shawinigan aluminum smelter

PAHs were grouped based on their volatility (volatile: acenaphthene, fluorine, phenanthrene, anthracene; semi-volatile: fluoranthene, pyrene, retene, benz(a)anthracene, chrysene; and particle-associated: benzo(b)fluoranthene, benzo(k)fluoranthene, benzo(a)pyrene, indeno(123-cd)pyrene, dibenz(a,h)anthracene, benzo(ghi)perylene) and compared across sites and size fractions (Fig. 2). Semi-volatile and particle-associated PAHs tended to be enriched in the fine and coarse fractions collected near metal industry sites in Hamilton (HB), Montreal (MC), and Shawinigan (SW) compared to petrochemical sites in Sarnia (SR) and Montreal (MA). Volatile PAHs tended to be enriched in the coarse and super-coarse fractions across most sites with the exception of Shawinigan, which displayed the highest levels of semi-volatile and particle-associated PAHs in these fractions.

### Cytotoxic responses

Particle-induced cytotoxicity was assessed across a range of doses by measuring resazurin reduction, an indicator of metabolic activity, and LDH release, an indicator of membrane permeability, in two cell lines: macrophage-like J774A.1 cells and A549 lung epithelial-like cells (Additional files 5, 6, 7 and 8, left panels). Particles produced dose–response relationships across the sites and size-fractions (*SIZE* x *DOSE* interaction, *p* < 0.05 for all assays; see figure legends for statistical analyses). Effects of particles differed across sites, however, as revealed by significant *SITE* x *SIZE* and *SITE* x *DOSE* interactions (*p* < 0.05). In contrast, exposure to extracts of field blank filters produced minimal response across cell lines and assays (Additional files 5, 6, 7 and 8, right panels) with the exception of the LDH response in A549 cells in which the blank filter extracts produced a small but significant dose-related response. In this cell line the UFP and PM_0.1–2.5_ fractions did not elicit a significantly greater LDH response than was observed for the corresponding blank filter extracts; however, PM_2.5–10_ and PM_>10_ samples produced a markedly greater response compared to blank filter extracts.

**Fig. 3:**
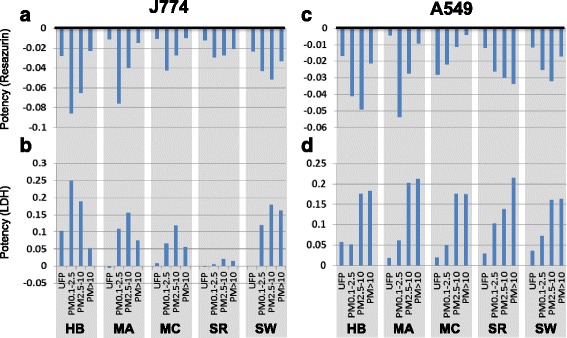
Cytotoxic potency of size-fractionated particles from industrial sites. Cytotoxic potency (β) was estimated using the slope of the dose-effect relationship from *n* = 3 independent experiments as described in the Methods section. Potency was assessed according to resazurin reduction and lactate dehydrogenase (LDH) release in J774A.1 macrophage-like cells (**a**, **b**) and A549 epithelial-like cells (**c**, **d**) respectively. For both assays, the height of the bar represents the potency, with greater negative numbers in the resazurin reduction assay and greater positive numbers in the LDH release assay corresponding to increased potency. HB, Hamilton Beach steel mill; MA, Montréal petrochemical refinery; MC, Montréal copper smelter; SR, Sarnia petrochemical refinery; SW, Shawinigan aluminum smelter

To reduce complexity and facilitate comparison of the pattern of effects, cytotoxic potencies according to resazurin reduction and LDH release assays were estimated using the slope of dose–response relationships observed for each assay. Potency estimates for both J774A.1 and A549 cells varied considerably across sites and sizes (Fig. 3). The pattern of potency estimates in J774A.1 cells was generally consistent for resazurin reduction and LDH assays (*r* = 0.78, *p* < 0.001). Sarnia particles of all size fractions exhibited low potency according to both cytotoxicity assays in J774A.1 cells compared to particles from other sites (Fig. 3a, b). The ultrafine fraction produced negligible cytotoxicity in resazurin reduction and LDH assays, with the exception of Hamilton and Shawinigan UFP which produced modest cytotoxic responses. For the remaining size fractions, potency estimates were variable across sites, with the fine (PM_0.1–2.5_) or coarse (PM_2.5–10_) fractions generally more potent than the super-coarse (PM_>10_) fraction. Whereas fine, coarse, and super-coarse particles from Hamilton steel, Montréal petrochemical, and Montréal copper exhibited significant size-dependent contrasts in potency according to both cytotoxic assays, Sarnia petrochemical and Shawinigan aluminum site particles tended to exhibit more similar potency across size-fractions.

Exposure of A549 cells produced a similar profile of resazurin reduction (Fig. 3c) to that observed in J774A.1 cells (*r* = 0.84, *p* < 0.001). In contrast, the pattern of LDH release in A549 cells (Fig. 3d) differed significantly from the pattern of resazurin reduction in the same cells (*r* = 0.23, *p* = 0.34) and with the LDH response in J774A.1 cells (*r* = 0.33, *p* = 0.16), with less evidence of site-dependent differences and more uniform size-dependent effects. For example, whereas Sarnia particles exhibited little capacity to provoke LDH release in J774A.1 cells (Fig. 3b), in A549 cells the LDH profile was similar to that seen for other sites (Fig. 3d). Potency measured by A549 LDH release tended to increase with particle size (UFP ≤ PM_0.1–2.5_ < PM_2.5–10_ ≤ PM_<10_).

### Cytokine release

**Fig. 4:**
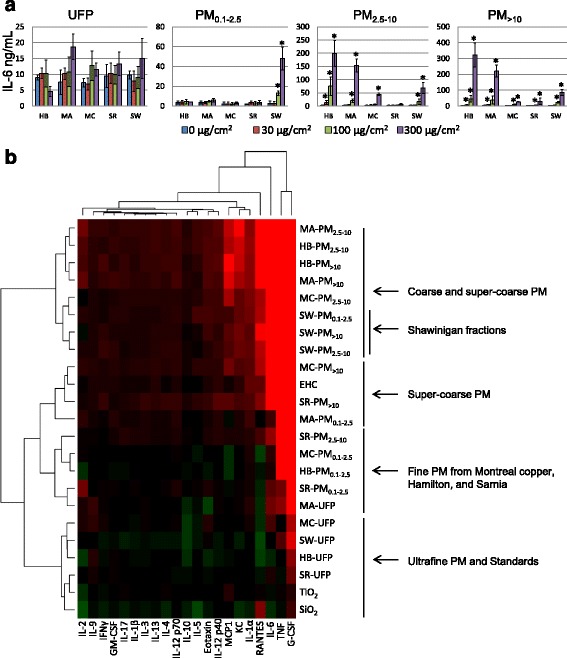
Inflammatory response to particles collected at industrial sites. Cytokine responses were measured in cell culture supernatants of J774A.1 cells exposed to size-fractionated particles (*n* = 3 independent experiments). **a** Interleukin (IL)-6 levels released by J774A.1 cells in response to a 24 h exposure to UFP, PM_0.1–2.5_, PM_2.5–10_ and PM_>10_ from industrial sites. Three-way ANOVA, *Dose x Site x Size* interaction, *p* = 0.002. For simplicity, only significant dose effects relative to 0 μg/cm^2^ within a *Site* and *Size* are presented (*p < 0.05, Holm-Sidak). **b** Hierarchical clustering of particles according to cytokine response in J774A.1 cells. The heat map displays particle potency estimates determined for each cytokine from the slope of the dose-effect relationship. General descriptions of the clusters are presented on the right. Red, increased expression; green, decreased expression. HB, Hamilton Beach steel mill; MA, Montréal petrochemical refinery; MC, Montréal copper smelter; SR, Sarnia petrochemical refinery; SW, Shawinigan aluminum smelter

The inflammatory potential of particles was assessed by measuring cytokine levels released by J774A.1 cells into cell culture media. In contrast to exposures to field blank filter extracts (Additional file 9), particle exposures altered cytokine levels in a dose-dependent manner characterised by clear size- and site-dependent effects (Fig. 4). For example, IL-6 responded strongly to the PM_2.5–10_ and PM_>10_ size-fractions, but less so to the fine PM_0.1–2.5_ fraction with the exception of Shawinigan PM_0.1–2.5_ (*Site* x *Size* x *Dose* interaction, *p* = 0.002; Fig. 4a). To facilitate comparison of inflammatory signaling across samples, particle inflammatory potency was estimated using the slope of the dose–response curve for each cytokine. Two-way clustering of cytokines and samples according to potency for the most part separated particles by size, with some evidence of clustering according to site (Fig. 4b). Hamilton and Montréal petrochemical site PM_2.5–10_ and PM_>10_ samples formed a cluster characterised by strong inflammatory response, characterised by markedly higher levels of IL-6, RANTES, MCP-1, IL-1α, and KC. Sarnia and Montréal copper refinery PM_>10_ samples also produced a strong response, and clustered with the urban standard EHC-93 and with Montréal petrochemical site PM_0.1–2.5_. The remaining fine PM_0.1–2.5_ fractions (Montréal copper refinery, Hamilton steel, Sarnia petrochemical) clustered together and were generally of lower potency. The two mineral dusts TiO_2_ and SiO_2_, included as standard reference materials, clustered together with most UFP. TiO_2_ elicited little inflammatory response, while cytokine release tended to decrease with increasing dose of SiO_2_. UFP exposure produced a unique profile of cytokine response compared to other particles, characterised by decreased IL-10 (*Size* x *Dose* interaction, *p* < 0.001; *Site* x *Size* interaction, *p* = 0.035) and RANTES (*Site* x *Size* x *Dose* interaction, *p* = 0.025), as well as a modest increase of G-CSF (*Site* x *Size* x *Dose* interaction, *p* < 0.001). In contrast to the size-dependent effects, three Shawinigan samples (PM_0.1–2.5_, PM_2.5–10_, PM_>10_) elicited a similar inflammatory response and clustered together, while Shawinigan UFP tended to decrease cytokine expression to a greater extent than other sites.

### Integrated estimate of potency

**Fig. 5:**
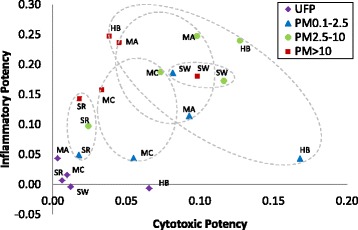
Integrated assessment of particle potency as a function of cytotoxic and inflammatory potency estimates. Potency estimates were calculated for responses of J774. A1 cells to particles described in the Methods. Groupings of particles (excluding UFP) from the same site are indicated by dashed lines. HB, Hamilton Beach steel mill; MA, Montréal petrochemical refinery; MC, Montréal copper smelter; SR, Sarnia petrochemical refinery; SW, Shawinigan aluminum smelter

Contrasts in particle effects across sites and sizes may be better visualised by integrating particle potency assessed by cytotoxicity and inflammatory measures [12]. As J774A.1 cells appeared to be more sensitive to site-dependent differences, integrated potency estimates were generated for responses measured using cytotoxic (resazurin reduction, LDH release) and inflammatory (mean of all cytokines) responses in these cells. Plotting cytotoxic and inflammatory potency for all particles (Fig. 5) showed that UFP exhibited the lowest cytotoxic and inflammatory potency, with the exception of Hamilton UFP, which exhibited relatively high cytotoxic potency but little inflammatory potency. All remaining size fractions from Shawinigan clustered together (relatively high cytotoxic and inflammatory potential), as did fractions from Sarnia (relatively low cytotoxic and inflammatory potential). Particles from the two Montréal sites generally clustered together, with fractions from the petrochemical site tending to have slightly higher cytotoxic and inflammatory potency than the corresponding size fraction from the copper refinery site across size fractions. In contrast to these groupings, potency estimates for particles from Hamilton varied considerably across size fractions and were distributed around the periphery of the plot, exhibiting high cytotoxic/low inflammatory potency (fine PM_0.1–2.5_), high cytotoxic and inflammatory potency (PM_2.5–10_), or low cytotoxic/high inflammatory potency (PM_>10_).

### Regressions against total metals, PAH, and endotoxin content

**Fig. 6:**
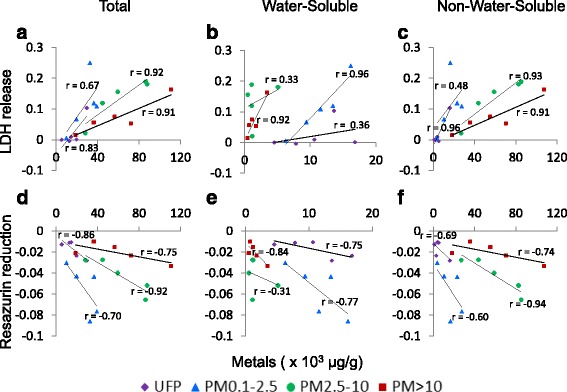
Association of cytotoxic potency in J774A.1 cells with metal content in size-fractionated particles. Cytotoxic potencies according to lactate dehydrogenase (LDH) release and resazurin reduction were regressed against total, water-soluble, and non-water-soluble metals. Pearson product–moment correlation coefficient r-values are presented. LDH release. **a** Total metals. UFP, *r* = 0.83, *p* = 0.08; PM_0.1–2.5,_ *r* = 0.67, *p* = 0.22; PM_2.5–10_, *r* = 0.92, *p* = 0.03; PM_>10_, *r* = 0.91, *p* = 0.03. **b** Water-soluble metals. UFP, *r* = 0.36, *p* = 0.55; PM_0.1–2.5_, *r* = 0.96, *p* < 0.01; PM_2.5–10_, *r* = 0.33, *p* = 0.59; PM_>10_, *r* = 0.92, *p* = 0.02. **c** Non-water-soluble metals. UFP, *r* = 0.96, *p* = 0.01; PM_0.1–2.5_, *r* = 0.48, *p* = 0.42; PM_2.5–10_, *r* = 0.93, *p* = 0.02; PM_>10_, *r* = 0.91, *p* = 0.03. Resazurin reduction. **d** Total metals. UFP, *r* = −0.86, *p* = 0.06; PM_0.1–2.5_, *r* = −0.70, *p* = 0.19; PM_2.5–10_, *r* = −0.92, *p* = 0.02; PM_>10_, *r* = −0.75, *p* = 0.15. **e** Water-soluble metals. UFP, *r* = −0.75, *p* = 0.14; PM_0.1–2.5_, *r* = -0.77, *p* = 0.13; PM_2.5–10_, *r* = −0.31, *p* = 0.61; PM_>10_, *r* = −0.84, *p* = 0.07. **f** Non-water-soluble metals. UFP, *r* = −0.69, *p* = 0.20; PM_0.1–2.5_, *r* = −0.60, *p* = 0.28; PM_2.5–10_, *r* = −0.94, *p* = 0.02; PM_>10_, *r* = −0.74, *p* = 0.15

The observation that plotting particle potency according to average cytotoxic and inflammatory responses grouped particles of similar composition (e.g., Shawinigan particles) suggested that composition, in addition to size, is an important determinant of potency. To explore the link between particle composition and in vitro response, potency estimates were regressed against metals (total, water-soluble, non-water-soluble), PAHs, and endotoxin content. In J774A.1 cells, metal content within each size fraction tended to be associated with potency according to both LDH release and resazurin reduction, but associations differed according to metal solubility (Fig. 6). Ultrafine particles exhibited little potency, and associations with metal content were driven by the response to the Hamilton sample. For the remaining size fractions, the slope describing the association between total metals and potency tended to decrease with increasing particle size (Fig. 6a, d), and mirrored the trends observed in the non-water-soluble fraction (Fig. 6c, f). In general, the potency of PM_0.1–2.5_ particles was more closely associated with water-soluble metals (Fig. 6b, e) while the potency of PM_2.5–10_ was more closely associated with non-water-soluble metals (Fig. 6c, f); associations between PM_>10_ potency and metals tended to be independent of solubility. Association of cytotoxic response with metals were generally weaker and non-significant in A549 cells (Additional file 10). Inflammatory potential in J774A.1 cells was positively correlated with total metals (*r* = 0.74, *p* < 0.001), non-water-soluble metals (*r* = 0.80, *p* < 0.001), and negatively correlated with water-soluble metals (*r* = −0.67, *p* = 0.001) when data from all size fractions were included. However, there was little evidence for associations between metals and inflammatory potential when size fractions were assessed individually (data not shown).

Total PAH content was associated with LDH release in J774A.1 cells (*r* = 0.52, *p* = 0.045) when all size fractions were included in the analysis. With the exception of a marginal association between PAHs in the PM_>10_ fraction and LDH release (*r* = 0.81, *p* = 0.09), associations were weak and non-significant when analysed within each size fraction (Additional file 11). Stratification of PAHs according to volatility showed that while cytotoxic responses were not associated with volatile PAH content, LDH release was associated with super-coarse fraction semi-volatile PAHs (*r* = 0.92, *p* = 0.03; *r* = 0.46, *p* = 0.09 when including data for all size fractions) and PM-associated PAHs (*r* = 0.85, *p* = 0.07; *r* = 0.63, *p* = 0.01 when including data for all size fractions). Resazurin reduction and inflammatory potential were not consistently associated with PAH content (Additional file 11).

Endotoxin was most strongly associated with inflammatory potency (J774A.1, *r* = 0.85, *p* < 0.001) and LDH release (J774A.1, *r* = 0.55, *p* = 0.01; A549, *r* = 0.71, *p* < 0.001), but not with resazurin reduction (J774A.1, *r* = 0.40, *p* = 0.08; A549, NS) when all size fractions were evaluated together. When size fractions were evaluated individually, the only significant association was with inflammatory potency in the PM_2.5–10_ fraction (*r* = 0.94, *p* = 0.02, Additional file 11).

### Regressions against specific metals

Given the overall positive correlations between metals and cytotoxicity endpoints, best subset regressions were performed on metal composition to identify potential drivers of toxicity. Analyses were performed on abundant metals (those exceeding 100 μg/g in at least one particle size/sample) in the water-soluble fraction (Al, V, Mn, Fe, Ni, Cu, Zn, As, Sr, Cd, Ba, Pb) and non-water-soluble fraction (Al, Ti, V, Cr, Mn, Fe, Ni, Cu, Zn, Sr, Sn, Ba, Pb). UFP were excluded from these analyses as they did not exhibit strong contrasts in potency across sites. Potency according to resazurin reduction in J774A.1 cells could be predicted by the linear combination of the concentration of water-soluble Mn, Fe, Cu, Zn, and Pb (β = 0.0168 + [0.000155 x Mn] – [0.0000178 x Fe] – [0.0000547 x Cu] – [0.0000156 x Zn] + [0.000246 x Pb]; r^2^ = 0.90, *p* < 0.05 for all elements). However, water-soluble Mn alone was sufficient to explain a significant proportion of the variance in potency (β = 0.0235 + [0.0000634 x soluble Mn]; r^2^ = 0.75, *p* < 0.001). Resazurin reduction in A549 cells was also most closely associated with Mn (β = 0.0189 + [0.0000317 x soluble Mn]; r^2^ = 0.47, *p* = 0.005). LDH release in J774A.1 cells was predicted by a linear combination of water-soluble manganese and iron (β = 0.0684 + [0.000334 x soluble Mn] – [0.0000676 x soluble Fe]; r^2^ = 0.69, Mn *p* = 0.002, Fe *p* = 0.011), with manganese again being the best single predictor (β = 0.0743 + [0.000122 x soluble Mn]; r^2^ = 0.26, *p* = 0.05). No soluble element predicted LDH release in A549, although there was a significant inverse relationship with nickel (β = 0.198 – [0.000860 x soluble Ni]; r^2^ = 0.81, *p* < 0.001).

Regressing against non-water-soluble metal levels, resazurin reduction in J774A.1 cells was predicted by a linear combination of lead, zinc, and strontium (β = 0.023 + [0.000145 x Pb] + [0.0000127 x Zn] – [0.0000799 x Sr]; r^2^ = 0.90, Pb *p* < 0.001, Zn *p* = 0.013, Sr *p* < 0.001), which could be simplified to lead alone (β = 0.0176 + [0.000155 x Pb]; r^2^ = 0.571, *p* = 0.001). In A549 cells, resazurin reduction was predicted by the concentration of non-water-soluble lead, iron, and strontium (β = 0.0192 + 0.0000696 x Pb] + [0.000000322 x Fe] – [0.0000548 x Sr]; r^2^ = 0.73, Pb *p* = 0.006, Fe *p* = 0.055, Sr *p* = 0.002). Lactate dehydrogenase release in J774A.1 cells was predicted by a linear combination of non-water-soluble lead, zinc, and aluminium (β = −0.0151 x [0.000381 x Pb] + [0.0000526 x Zn] + [0.00000158 x Al], r^2^ = 0.698, Pb *p* = 0.008, Zn *p* = 0.040, Al *p* = 0.012], and in A549 cells by non-water-soluble titanium and tin (β = 0.146 + [0.0000647 x Ti] – [0.00119 x Sn]; r^2^ = 0.60, Ti *p* = 0.004, Sn *p* = 0.022].

## Discussion

Industrial emissions are important contributors to particulate air pollution levels, and may contribute to geographical disparities in the health impacts of particulate matter identified in epidemiological studies [3, 6, 7]. There has, however, been little direct comparison of the toxicity of particles from multiple industrial sources. In the present study, particles collected in the vicinity of industrial sources were compared across size fractions on an equal mass basis, across a range of doses, in two cell lines, using a panel of cytotoxic and inflammatory assays to characterise the profile of response. Our results demonstrate that particles collected near distinct industrial sites display strong contrasts in composition and in vitro toxicity, substantiating the hypothesis that industrial sources differentially impact the toxicity of airborne particulate matter.

The distinct composition of particles collected in the vicinity of industrial sites, notably with respect to metals, suggests that emissions from dominant local sources were enriched in our samples collected over a period of weeks, as expected. All size-fractions displayed differences in the total concentration of metals, but the extent to which constituent metals varied depended on the size-fraction. Metal composition of coarse (PM_2.5–10_) and super-coarse (PM_>10_) fractions was similar across sites, consisting mostly of the common crustal elements aluminium and iron. In contrast, UFP and fine (PM_0.1–2.5_) particles exhibited considerable variability across sites generally consistent with significant impact of the local sources targeted by our sampling (e.g., elevated zinc, manganese and arsenic near the Hamilton steel mills; elevated copper, arsenic, and lead near the Montréal copper refinery; elevated aluminum near the Shawinigan aluminium smelter; [31, 32]). Relatively high levels of copper were also detected at the Montréal petrochemical site, itself downwind of the copper refinery, illustrating how multiple sources can contribute to the composition of particles collected at a given site, particularly in a complex location like East Montréal. Contrasts were especially evident in the water-soluble fraction, which represented a significant proportion of total metals in ultrafine and fine particles (45 ± 13% and 70 ± 17% average water-solubility respectively compared to 2–3% average water-solubility of metals in the coarse fractions). While most sites displayed considerable variability in composition across size ranges, size-fractionated particles in Shawinigan were remarkably similar, with aluminium being the dominant metal in all samples. Importantly, the data show that differences in metal composition were most prominent in the water-soluble fraction of ultrafine and fine particles, suggesting that industrial emissions can significantly impact levels of bioavailable elements in respirable particles. Semi-volatile and particle-associated PAHs appeared to be enriched in either or both of the fine and coarse particle fractions collected near metal industry sites in Hamilton (HB), Montréal (MC), and Shawinigan (SW) compared to petrochemical industry sites in Montréal (MA) and Sarnia (SR), suggesting inter-site differences in the organic fraction. The presence of volatile PAHs in the coarse and super-coarse fractions may represent absorption onto the collection substrate rather than particle composition [33], as a significant fraction will be in the gas-phase. Collectively, the data confirm that our sampling strategy resulted in the collection of particles of distinct composition related to specific source emissions.

The clear inter-site variability in particle composition provided a basis for examining to what extent source-dependent differences in particle composition altered the potency of particles in biological models. While a number of studies have compared the potency of ambient particles collected in different sites, including urban vs. rural [11] and residential vs. traffic vs. industrial [10, 12], there has been little direct comparison of the toxicity of particles collected at multiple industrial sites. Several key observations were made in the present study. First, particles collected in the vicinity of industrial sites displayed striking differences in overall potency. For example, particles collected near Hamilton steel industry tended to be among the most potent across size fractions, whereas particles collected near Sarnia petrochemical industry were among the least potent. Second, particle potency appeared to be a function of both particle size and particle composition. Whereas samples from Hamilton steel, Montréal petrochemical, and Montréal copper sites exhibited contrasting composition and potency across size fractions within each site, particles collected in the vicinity of the Shawinigan aluminium refinery that were very similar in composition (predominantly aluminium) tended to exhibit similar potency across size-fractions. Third, the cytotoxic and inflammatory potential of particle samples was associated with the levels of specific constituents (metals, PAHs, endotoxin). Fourth, effects differed across cell lines and assays, suggesting differential responsiveness to particle constituents. The contrasting effects of particles compared on an equal mass basis is consistent with the hypothesis that particles collected in the vicinity of distinct sources will exhibit a range of cytotoxic and inflammatory potencies. Collectively, these observations suggest that particle potency is influenced both by particle size and composition, with biological responses being a function of particle properties and cell characteristics.

While coarse particles generally represent a significant proportion of total particle mass, they are less efficiently deposited in the alveolar regions of the lungs and may contain less bioavailable metal content. In contrast, smaller particles may be present in greater number, have a greater reactive surface:volume ratio per unit mass, and tend to contain a greater concentration of soluble metals, characteristics that may increase their potency in vivo. Despite these differences between fine and coarse particles, the coarse fraction of ambient particles can exert relatively high cytotoxic and inflammatory effects in cell culture models or when instilled into the lungs of experimental animals compared to ultrafine and fine particles [12, 17, 19, 34–36]. We observed size-dependent effects on both cytotoxicity and inflammatory potential in our cell culture models: fine and coarse particles tended to be more cytotoxic, whereas coarse and super-coarse particles tended to be more inflammogenic. The cell culture models and assays employed in the present study were less sensitive to UFP than to larger size fractions in terms of the magnitude of response. There are several explanations beyond lower inherent potency of the UFP, including reduced particle-cell interaction resulting from size-dependent differences in phagocytosis by macrophages [37] or differences in particle settling rate that impact the effective dose and timing of effects. However, the effects of UFP on cytokine release confirms that these particles, while not acutely cytotoxic in the present model according to the resazurin reduction and LDH release assays, did impact biological function, and were therefore neither inert nor invisible to the cells.

The significant variability in response across industrial sites within each size fraction indicated that the cell culture models and assays employed were sensitive to particle characteristics other than particle size. Considerable attention has been directed towards elucidating determinants of particle potency, including investigation of the role of metals, PAHs, and endotoxin [35, 38]. Total metal content was associated with cytotoxic potency, and to a lesser extent inflammatory potency, while PAHs and endotoxin were associated with cytotoxicity and inflammatory potency respectively primarily in the coarse fractions. These results are, in a broad sense, consistent with previous observations by ourselves and others [9, 12, 19, 39]. Despite representing only a fraction of total particle mass, trace metal concentrations explained a significant proportion of the variance in cytotoxic potency. For example, Sarnia particles, which had the lowest metal content across size-fractions, also displayed the lowest cytotoxic potency, whereas the high metal content in the fine fraction of Hamilton, Montréal, and Shawinigan sites was associated with higher toxicity. These results, reflecting strong contrasts in particle composition as a result of sampling in the vicinity of important industrial point sources, are in line with a body of work linking trace metals with particle toxicity through in vitro, in vivo, and population studies [38]. Endotoxin levels were significantly higher in the coarse and super-coarse fractions, and likely account for a portion of the inflammatory and cytotoxic effects, consistent with other studies [9, 19]. While PAHs were not strongly linked to the biological indices evaluated here beyond correlations with LDH release in the super-coarse fraction, they may nevertheless contribute to the overall cytotoxic and inflammatory potency of fine and coarse particles [19, 36, 40].

There is considerable evidence that soluble metal content is an important determinant of pulmonary and systemic impacts of urban and combustion emission particles [13, 14, 16]. However, insoluble metals have also been linked to biological reactivity [18, 41], and effects of this fraction warrant attention given the relative abundance of insoluble metals. As significant associations were found for both water-soluble (fine fraction) and non-water-soluble metals (coarse fractions), it was not possible to unambiguously attribute effects to water-soluble or non-water-soluble metals. The slope of the relationship between non-water-soluble metals and potency appeared to decrease with increasing particle size, suggesting an interaction between physical and chemical characteristics of the particles. This could possibly reflect the actual effective internal dose experienced by cell (i.e., due to differential intake of material [42]), or interactions of independent physical and chemical effects associated with particle size. On the other hand, the concentration of active agents may vary with particle size: as particle size increases, crustal elements of lower solubility predominate and metals derived from anthropogenic sources are less abundant. Particle size likely influences both how cells experience metals (as a result of surface and endocytic interactions), as well as the nature of the metals to which cells are exposed (due to differences in particle composition and metal solubility), which may explain apparent changes in the potency of metals across size fractions.

Given that the size-dependency of the effects could be due to differential distribution of a toxicity determinant across size fractions, we performed a series of regression analyses to evaluate whether specific metals better explained the variance in particle potency. A number of models were generated that contained a variety of metals, with soluble manganese emerging as the factor most closely associated with cytotoxicity across size fractions, particularly with respect to resazurin reduction. Soluble manganese covaried closely with zinc and other metals across sites, and could be a surrogate for other elements, and so caution should be applied in attributing effects to any one element. Nevertheless, these associations are in a broad sense consistent with the overall coherence among in vitro, in vivo, and population studies implicating soluble transition metals in adverse health effects of ambient particles [14, 38, 40, 43]. While manganese is an essential element and deficiency is associated with adverse health impacts, chronic inhalation of manganese is neurotoxic, associated with both cognitive and motor deficits, and may have cardiovascular impacts [44]. Manganese chloride has been shown to inhibit cell proliferation and induce apoptosis in A549 cells [45], demonstrating in vitro toxicity. The acute toxicity resulting from intratracheal instillation of the urban particle standard EHC-93 was reproduced by administration of soluble zinc, implicating zinc in the toxicity of ambient particulate matter [13]. Consistent with a role for soluble zinc in mediating cytotoxic effects of particle exposure, the in vitro cytotoxicity of zinc oxide nanoparticles appears to be at least partly due to levels of free zinc ions [46]. Thus, while associations made on the basis of a small number of samples should be interpreted with caution, the associations appear consistent with the established toxicity of soluble transition metals. It is important to note that the relatively high concentrations of transition metals in UFP compared to other size fractions did not correlate well with cytotoxicity, suggesting that soluble metal content alone does not explain the toxicity of the particles. The failure to find specific metal drivers of LDH release across size fractions may relate to a greater sensitivity of this assay to particle characteristics or constituents (e.g., endotoxin) that co-vary with size, as LDH release correlated reasonably well with metals when analysed within each size fraction.

The J774A.1 macrophage-like and A549 epithelial-like cell lines displayed certain differences in their responses to particle size and constituents according to the cytotoxicity assays (resazurin reduction, LDH release) employed. J774A.1 cells appeared to be sensitive to metal composition in addition to particle size, and although not statistically significant in all comparisons, the relationship appeared to hold across particle sizes. In contrast, A549 cells appeared to be more sensitive to particle size than to particle composition. This was particularly evident in the similar size-dependent effects of particles on A549 LDH release across sampling sites. These differences between cell lines are consistent with our previous observations using these models [12], and with other comparisons across cell lines [47]. Macrophage-like J774A.1 cells are capable of phagocytising particles at a high rate and thus possibly experience a greater internal dose of metals than A549 cells, which may explain their apparently higher sensitivity to differences in metal composition. It is noteworthy that had we employed only A549 cells, commonly used to assess particle toxicity, and LDH release, an established cytotoxicity assay, we would have likely concluded that there was little evidence of differences in the toxic potency of particles collected in the vicinity of industrial sites. Our findings reinforce the importance of using multiple cell lines and assays to assess particle toxicity, as different cells and assays may be sensitive to distinct particle characteristics [12].

The primary objective of the present work was to evaluate to what extent particles collected in the vicinity of different industrial sources exhibit contrasts in composition and potency. Strengths of the present study include direct comparison of size-fractionated particles with contrasting chemical composition across a range of doses, employment of two cell lines, and assessment of toxic potency using a panel of cytotoxic and inflammatory assays. However, certain factors should be considered when interpreting results. While samples were collected as close as possible to each industrial site, some differences between the locations could be due to seasonal influences. For example, the amount and composition of regionally-transported particles is seasonally dependent, and their variable influence could add noise to the analysis of differential biological responses as has been shown previously [48, 49]. A limitation common to this type of toxicological analysis of environmental samples is that the extracted material will likely differ from the ambient aerosols at the time of sampling. The aqueous extraction of particles, while reasonably efficient at extracting mass from polyurethane foam and less efficient from polypropylene filters, likely modified the composition of the extracted materials compared to the composition of total particulates suspended in the air at the five sampling sites we studied. Poor extraction of non-polar organic constituents may explain the contrast in PAH composition measured in the present work compared with analyses performed using solvent- and acid-based extractions on other urban ambient particles (e.g., [12]). It is also likely that a proportion of volatile and semi-volatile PAHs were lost during the extraction process. In addition, some of the semi-volatile PAHs measured may have been due to the capture of gas phase compounds by the PUF material as opposed to originating on particles [33]. Accordingly, while performing chemical and toxicological analyses on the same extracted material facilitated the association of particle constituents to biological effects, it is clear that the chemical profile of extracted particles to which cells were exposed may differ from that inhaled from ambient air. Nevertheless, the metal composition of the particles is consistent with expected enrichment of source emissions, and the higher PAH levels measured at the Shawinigan site is consistent with aluminium smelters being a significant anthropogenic source of PAHs released to the atmosphere. The cytotoxic and inflammatory indices assessed here were selected to represent distal endpoints that integrate the cellular response as a whole to multiple physical and chemical stressors. While measurement of biological effects at a single time point allowed comparison across sites and particle sizes, the pattern of effects may differ at other time points (e.g., due to differential kinetics of response of individual cytokines). Assays that target specific particle components or enable assessment of the activation of specific biological pathways may identify other exposure and mechanistic signatures [8, 28].

## Conclusions

This study confirms that local industrial sources are important producers of particulate constituents that differentially impact biological function and that differ depending on industrial processes. Validation of these findings using multiple complementary assays, manipulation of particle constituents, and blockade of biological pathways should help verify the relative importance of identified factors in driving such effects. Attribution of toxic potency to specific particle constituents will provide critical data for identification of priority sources for regulatory action.

## Additional files

Additional file 1: Metal composition of all size-fractionated particles. Table of metal content according to particle size and sampling site. (XLSX 35 kb)
Additional file 2: PAH composition of all size-fractionated particles. Table of PAH content according to particle size and sampling site. (XLSX 15 kb)
Additional file 3: Clustering of particulate matter samples according to metal content. Size-fractionated particles collected in the vicinity of industrial sites were clustered according to A) water-soluble metals, B) total metals, and C) non-water-soluble (NWS) metals (average linkage, Pearson correlation coefficient distance). (PDF 200 kb)
Additional file 4: Clustering of elements according to water soluble, non-water- soluble and total elements. A) Water-soluble (WS), B) non-water soluble (NWS) and C) total metals were clustered to reveal the associations (covariance) among sets of elements. (PDF 22 kb)
Additional file 5: Cytotoxic responses of J774A.1 cells to 24 h exposure to size-fractionated particulate matter collected in the vicinity of industrial sites according to the resazurin reduction assay. Metabolic reduction of non-fluorescent resazurin in J774A.1 cells exposed for 24 h to size-fractionated and standard reference particles (left side) and extracts from corresponding field blank filters that were transported to each site but remained unexposed to ambient air (right side). Values are presented as average fold-effect (FE) over control ± standard error (*n* = 3 independent experiments). HB, Hamilton Beach steel mill; MA, Montréal petrochemical refinery; MC, Montréal copper smelter; SR, Sarnia petrochemical refinery; SW, Shawinigan aluminum smelter. Size-fractionated and standard reference particles. Three-way ANOVA (Size-fractionated particles): *Site* x *Dose* (*p* = 0.002) and *Size* x *Dose* (*p* < 0.001) interactions. Asterisks represent significant pairwise comparisons (Holm-Sidak) as follows: doses within *Site* significantly different from 0 μg/cm^2^, or sites within *Size* significantly different from one another as indicated by brackets (**p* < 0.05, ***p* < 0.001). Letters (a-e) represent sizes within *Site* significantly different from one another (*p* < 0.05). Two-way ANOVA (Standards): *Particle* (*p* < 0.001) and *Dose* (*p* < 0.001) main effects. Asterisks represent significant pairwise comparisons (Holm-Sidak) as follows: doses significantly different from 0 μg/cm^2^, or particles significantly different from one another as indicated by brackets (**p* < 0.05, ***p* < 0.001). Field blanks. Three-way ANOVA: *Site* x *Size* (*p* = 0.05) interaction. No significant pairwise comparisons (Holm-Sidak) were observed. (PDF 52 kb)
Additional file 6: Cytotoxic responses of J774A.1 cells to 24 h exposure to size-fractionated particulate matter collected in the vicinity of industrial sites according to the LDH release assay. Lactate dehydrogenase (LDH) release into cell culture supernatants of J774A.1 cells exposed for 24 h to size-fractionated and standard reference particles (left-side) and extracts from corresponding field blank filters that were transported to each site but remained unexposed to ambient air (right side). Data represent LDH release adjusted for total cellular LDH content. Values are presented as average fold-effect (FE) over control ± standard error (*n* = 3 independent experiments). HB, Hamilton Beach steel mill; MA, Montréal AIEM petrochemical refinery; MC, Montréal copper smelter; SR, Sarnia petrochemical refinery; SW, Shawinigan aluminum smelter. Size-fractionated and standard reference particles. Three-way ANOVA (Size-fractionated particles): *Site* x *Dose* (*p* < 0.001) and *Size* x *Dose* (*p* < 0.001) interactions. Asterisks represent significant pairwise comparisons (Holm-Sidak) as follows: doses within *Size* significantly different from 0 μg/cm^2^ (**p* < 0.05, ***p* < 0.001). Letters (a-g) represent doses within *Site* significantly different from 0 μg/cm^2^ (*p* < 0.05). Bars with the same symbol (#,@,&,^,$) indicate sites within *Dose* that are significantly different from one another (*p* < 0.05). Two-way ANOVA (Standards): *Particle* (*p* < 0.001) and *Dose* (*p* < 0.001) main effects. Asterisks represent significant pairwise comparisons (Holm-Sidak) as follows: doses significantly different from 0 μg/cm^2^, or particles significantly different from one another as indicated by brackets (**p* < 0.05, ***p* < 0.001). Field blanks. Three way ANOVA: *Size* x *Dose* (*p* = 0.021) interaction. Asterisks represent doses within *Size* significantly different from 0 μg/cm^2^ (**p* < 0.05). Letters (a-c) represent sizes within *Dose* significantly different from one another (*p* < 0.05). (PDF 55 kb)
Additional file 7: Cytotoxic responses of A549 cells to 24 h exposure to size-fractionated particulate matter collected in the vicinity of industrial sites according to the resazurin reduction assay. Metabolic reduction of non-fluorescent resazurin in A549 cells exposed for 24 h to size-fractionated and standard reference particles (left-side) and extracts from corresponding field blank filters that were transported to each site but remained unexposed to ambient air (right-side). Values are presented as average fold-effect (FE) over control ± standard error (*n* = 3 independent experiments). HB, Hamilton Beach steel mill; MA, Montréal AIEM petrochemical refinery; MC, Montréal copper smelter; SR, Sarnia petrochemical refinery; SW, Shawinigan aluminum smelter. Size-fractionated and standard reference particles. Three-way ANOVA (Size-fractionated particles): *Site* x *Size* (*p* < 0.001) and *Size* x *Dose* (*p* = 0.019) interactions. Asterisks represent significant pairwise comparisons (Holm-Sidak) as follows: doses within *Site* significantly different from 0 μg/cm^2^, or sites within *Size* significantly different from one another as indicated by brackets (**p* < 0.05, ***p* < 0.001). Letters (a-h) represent sizes within *Site* significantly different from one another (*p* < 0.05). Two-way ANOVA (Standards): *Particle* x *Dose* interaction (*p* = 0.012). Asterisks represent significant pairwise comparisons (Holm-Sidak) as follows: doses within *Particles* significantly different from 0 μg/cm^2^ (***p* < 0.001). Letters (a,b) represent sites within *Dose* significantly different from one another (*p* < 0.5). Field blanks. Three-way ANOVA: *Site* x *Size* (*p* < 0.001) interaction. Asterisks represent significant pairwise comparisons (Holm-Sidak) as follows: sites within *Size* significantly different from one another as indicated by brackets (**p* < 0.05, ***p* < 0.001). Letters (a-e) represent sizes within *Site* significantly different from one another (*p* < 0.05). (PDF 54 kb)
Additional file 8: Cytotoxic responses of A549 cells to 24 h exposure to size-fractionated particulate matter collected in the vicinity of industrial sites according to the LDH release assay. Lactate dehydrogenase (LDH) release into cell culture supernatants of A549 cells exposed for 24 h to size-fractionated and standard reference particles (left-side) and extracts from corresponding field blank filters that were transported to each site but remained unexposed to ambient air (right side). Data represent LDH release, adjusted for total cellular LDH content. Values are presented as average fold-effect (FE) over control ± standard error (*n* = 3). HB, Hamilton Beach steel mill; MA, Montréal AIEM petrochemical refinery; MC, Montréal copper smelter; SR, Sarnia petrochemical refinery; SW, Shawinigan aluminum smelter. Size-fractionated and standard reference particles. Three-way ANOVA (Size-fractionated particles): *Site* x *Size* (*p* = 0.002) and *Size* x *Dose* (*p* = 0.018) interactions. Asterisks represent significant pairwise comparisons (Holm-Sidak) as follows: doses within *Size* significantly different from 0 μg/cm^2^, or sites within *Size* significantly different from one another as indicated by brackets (**p* < 0.05, ***p* < 0.001). Letters (a-n) represent sizes within *Site* significantly different from one another (*p* < 0.05). Two-way ANOVA (Standards): *Particle* (*p* = 0.002) and *Dose* (*p* < 0.001) main effects. Asterisks represent significant pairwise comparisons (Holm-Sidak) as follows: doses significantly different from 0 μg/cm^2^, or particles significantly different from one another as indicated by brackets (**p* < 0.05, ***p* < 0.001). Field blanks. Three-way ANOVA: *Dose* (*p* < 0.001) main effect. Letter (a) spanned by a line represents doses significantly different from 0 μg/cm^2^ (*p* < 0.001). (PDF 55 kb)
Additional file 9: Inflammatory response to field blank filter extracts. Hierarchical clustering of particles according to cytokine response in J774A.1 cells exposed to field blank filter extracts. The heat map displays particle potency estimates determined for each cytokine from the slope of the dose-effect relationship. Red, increased expression; green, decreased expression. bHB, field blank from Hamilton Beach steel mill; bMA, field blank from Montréal petrochemical refinery; MC, field blank from Montréal copper smelter; SR, field blank from Sarnia petrochemical refinery; SW, field blank from Shawinigan aluminum smelter. (PDF 84 kb)
Additional file 10: Association of biological effects in A549 cells with metal content in size-fractionated particles. Cytotoxic potencies according to lactate dehydrogenase (LDH) release and resazurin reduction were regressed against total, water-soluble, and non-water-soluble metals. Pearson product–moment correlation coefficient r-values are presented. LDH release. A) Total metals. UFP, *r* = 0.77, *p* = 0.13; PM_0.1–2.5_, *r* = −0.55, *p* = 0.34; PM_2.5–10_, *r* = 0.32, *p* = 0.60; PM_>10_, *r* = −0.68, *p* = 0.21. B) Water-soluble metals. UFP, *r* = 0.51, *p* = 0.38; PM_0.1–2.5_, *r* = −0.64, *p* = 0.25; PM_2.5–10_, *r* = −0.35, *p* = 0.57; PM_>10_, *r* = −0.68, *p* = 0.20. C) Non-water-soluble metals. UFP, *r* = 0.75, *p* = 0.14; PM_0.1–2.5_, *r* = −0.46, *p* = 0.43; PM_2.5–10_, *r* = 0.36, *p* = 0.55; PM_>10_, *r* = −0.68, *p* = 0.21. Resazurin reduction. D) UFP, *r* = −0.19, *p* = 0.76; PM_0.1–2.5_, *r* = −0.63, *p* = 0.26; PM_2.5–10_, *r* = −0.60, *p* = 0.28; PM_>10_,*r* = 0.18, *p* = 0.78. Water-soluble metals. UFP, *r* = −0.20, *p* = 0.74; PM_0.1–2.5_, *r* = −0.41, *p* = 0.49; PM_2.5–10_, *r* = −0.09, *p* = 0.88; PM_>10_, *r* = 0.04, *p* = 0.95. Non-water-soluble metals. UFP, *r* = −0.12, *p* = 0.84; PM_0.1–2.5_, *r* = −0.65, *p* = 0.24; PM_2.5–10_, *r* = −0.62, *p* = 0.26; PM_>10_, *r* = 0.18, *p* = 0.77. (PDF 43 kb)
Additional file 11: Regressions of biological potency against total metals, water-soluble metals, non-water-soluble metals, endotoxin, and PAHs. Table of Pearson correlations and *p*-values. (PDF 71 kb)

## References

[1] Dockery DW, Pope CA, Xu X, Spengler JD, Ware JH, Fay ME, Ferris BG, Speizer FE (1993). An association between air pollution and mortality in six U.S. cities. N Engl J Med.

[2] Pope CA, Burnett RT, Thurston GD, Thun MJ, Calle EE, Krewski D, Godleski JJ (2004). Cardiovascular mortality and long-term exposure to particulate air pollution: epidemiological evidence of general pathophysiological pathways of disease. Circulation.

[3] Burnett RT, Brook J, Dann T, Delocla C, Philips O, Cakmak S, Vincent R, Goldberg MS, Krewski D (2000). Association between particulate- and gas-phase components of urban air pollution and daily mortality in eight Canadian cities. Inhal Toxicol.

[4] Crouse DL, Peters PA, van DA, Goldberg MS, Villeneuve PJ, Brion O, Khan S, Atari DO, Jerrett M, Pope CA (2012). Risk of nonaccidental and cardiovascular mortality in relation to long-term exposure to low concentrations of fine particulate matter: a Canadian national-level cohort study. Environ Health Perspect.

[5] Grahame TJ, Schlesinger RB (2007). Health effects of airborne particulate matter: do we know enough to consider regulating specific particle types or sources?. Inhal Toxicol.

[6] Zanobetti A, Franklin M, Koutrakis P, Schwartz J (2009). Fine particulate air pollution and its components in association with cause-specific emergency admissions. Environ Health.

[7] Peng RD, Dominici F, Pastor-Barriuso R, Zeger SL, Samet JM (2005). Seasonal analyses of air pollution and mortality in 100 US cities. Am J Epidemiol.

[8] Vincent R, Goegan P, Johnson G, Brook JR, Kumarathasan P, Bouthillier L, Burnett RT (1997). Regulation of promoter-CAT stress genes in HepG2 cells by suspensions of particles from ambient air. Fundam Appl Toxicol.

[9] Steenhof M, Gosens I, Strak M, Godri KJ, Hoek G, Cassee FR, Mudway IS, Kelly FJ, Harrison RM, Lebret E (2011). In vitro toxicity of particulate matter (PM) collected at different sites in the Netherlands is associated with PM composition, size fraction and oxidative potential—the RAPTES project. Part Fibre Toxicol.

[10] Dergham M, Lepers C, Verdin A, Billet S, Cazier F, Courcot D, Shirali P, Garcon G (2012). Prooxidant and proinflammatory potency of air pollution particulate matter (PM(2). (5)(−)(0). (3)) produced in rural, urban, or industrial surroundings in human bronchial epithelial cells (BEAS-2B). Chem Res Toxicol.

[11] Mirowsky J, Hickey C, Horton L, Blaustein M, Galdanes K, Peltier RE, Chillrud S, Chen LC, Ross J, Nadas A (2013). The effect of particle size, location and season on the toxicity of urban and rural particulate matter. Inhal Toxicol.

[12] Thomson EM, Breznan D, Karthikeyan S, MacKinnon-Roy C, Charland JP, Dabek-Zlotorzynska E, Celo V, Kumarathasan P, Brook JR, Vincent R (2015). Cytotoxic and inflammatory potential of size-fractionated particulate matter collected repeatedly within a small urban area. Part Fibre Toxicol.

[13] Adamson IY, Prieditis H, Hedgecock C, Vincent R (2000). Zinc is the toxic factor in the lung response to an atmospheric particulate sample. Toxicol Appl Pharmacol.

[14] Dreher KL, Jaskot RH, Lehmann JR, Richards JH, McGee JK, Ghio AJ, Costa DL (1997). Soluble transition metals mediate residual oil fly ash induced acute lung injury. J Toxicol Environ Health.

[15] Vincent R, Kumarathasan P, Goegan P, Bjarnason SG, Guenette J, Berube D, Adamson IY, Desjardins S, Burnett RT, Miller FJ et al.. Inhalation toxicology of urban ambient particulate matter: acute cardiovascular effects in rats. Res Rep Health Eff Inst. 2001;104:5–54. 11833973

[16] Wallenborn JG, Schladweiler MJ, Richards JH, Kodavanti UP (2009). Differential pulmonary and cardiac effects of pulmonary exposure to a panel of particulate matter-associated metals. Toxicol Appl Pharmacol.

[17] Schins RP, Lightbody JH, Borm PJ, Shi T, Donaldson K, Stone V (2004). Inflammatory effects of coarse and fine particulate matter in relation to chemical and biological constituents. Toxicol Appl Pharmacol.

[18] Imrich A, Ning Y, Kobzik L (2000). Insoluble components of concentrated air particles mediate alveolar macrophage responses in vitro. Toxicol Appl Pharmacol.

[19] Guastadisegni C, Kelly FJ, Cassee FR, Gerlofs-Nijland ME, Janssen NA, Pozzi R, Brunekreef B, Sandstrom T, Mudway I (2010). Determinants of the proinflammatory action of ambient particulate matter in immortalized murine macrophages. Environ Health Perspect.

[20] Pope CA, Schwartz J, Ransom MR (1992). Daily mortality and PM10 pollution in Utah Valley. Arch Environ Health.

[21] Frampton MW, Ghio AJ, Samet JM, Carson JL, Carter JD, Devlin RB (1999). Effects of aqueous extracts of PM(10) filters from the Utah valley on human airway epithelial cells. Am J Physiol.

[22] Jerrett M, Buzzelli M, Burnett RT, DeLuca PF (2005). Particulate air pollution, social confounders, and mortality in small areas of an industrial city. Soc Sci Med.

[23] Mattson ME, Guidotti TL (1980). Health risks associated with residence near a primary copper smelter: a preliminary report. Am J Ind Med.

[24] Pope CA, Rodermund DL, Gee MM (2007). Mortality effects of a copper smelter strike and reduced ambient sulfate particulate matter air pollution. Environ Health Perspect.

[25] Grahame TJ (2007). Mortality from copper smelter emissions circa 1967. Environ Health Perspect.

[26] Lippmann M, Ito K, Hwang JS, Maciejczyk P, Chen LC (2006). Cardiovascular effects of nickel in ambient air. Environ Health Perspect.

[27] Demokritou P, Kavouras IG, Ferguson ST, Koutrakis P (2002). Development of a high volume cascade impactor for toxicological and chemical characterization studies. Aerosol Sci Technol.

[28] Thomson EM, Williams A, Yauk CL, Vincent R (2009). Toxicogenomic analysis of susceptibility to inhaled urban particulate matter in mice with chronic lung inflammation. Part Fibre Toxicol.

[29] Fang T, Guo H, Verma V, Peltier RE, Weber RJ (2015). PM2.5 water-soluble elements in the southeastern United States: Automated analytical method development, spatiotemporal distributions, source apportionment, and implications for health studies. Atmos Chem Phys.

[30] Oakes MM, Burke JM, Norris GA, Kovalcik KD, Pancras JP, Landis MS (2016). Near-road enhancement and solubility of fine and coarse particulate matter trace elements near a major interstate in Detroit, Michigan. Atm Env.

[31] Jeong C-H, McGuire M, Herod D, Dann T, Dabek-Zlotorzynska E, Wang D, Ding L, Celo V, Mathieu D, Evans G (2012). Receptor model based identification of PM2.5 sources in Canadian cities. Atm Poll Res.

[32] Celo V, Dabek-Zlotorzynska E, Zereini F, Wiseman C (2010). Concentration and Source Origin of Trace Elements in PM2.5 Collected at Selected sites within the Canadian National Air Pollution Surveillance PM2.5 Speciation Program. Urban Airborne Particulate Matter: Origins,Chemistry, Fate and Health Impact.

[33] Galarneau E, Patel M, Brook JR, Charland J-P, Glasius R, Hung H: Artefacts in semivolatile organic compound sampling with polyurethane foam (PUF) substrates in high volume cascade impactors. Aerosol Science and Technology. 2016. In press.

[34] Becker S, Mundandhara S, Devlin RB, Madden M (2005). Regulation of cytokine production in human alveolar macrophages and airway epithelial cells in response to ambient air pollution particles: further mechanistic studies. Toxicol Appl Pharmacol.

[35] Schwarze PE, Ovrevik J, Hetland RB, Becher R, Cassee FR, Lag M, Lovik M, Dybing E, Refsnes M (2007). Importance of size and composition of particles for effects on cells in vitro. Inhal Toxicol.

[36] Monn C, Becker S (1999). Cytotoxicity and induction of proinflammatory cytokines from human monocytes exposed to fine (PM2.5) and coarse particles (PM10-2.5) in outdoor and indoor air. Toxicol Appl Pharmacol.

[37] Oberdorster G, Oberdorster E, Oberdorster J (2005). Nanotoxicology: an emerging discipline evolving from studies of ultrafine particles. Environ Health Perspect.

[38] Chen LC, Lippmann M (2009). Effects of metals within ambient air particulate matter (PM) on human health. Inhal Toxicol.

[39] Wang B, Li K, Jin W, Lu Y, Zhang Y, Shen G, Wang R, Shen H, Li W, Huang Y (2013). Properties and inflammatory effects of various size fractions of ambient particulate matter from Beijing on A549 and J774A.1 cells. Environ Sci Technol.

[40] Gerlofs-Nijland ME, Rummelhard M, Boere AJ, Leseman DL, Duffin R, Schins RP, Borm PJ, Sillanpaa M, Salonen RO, Cassee FR (2009). Particle induced toxicity in relation to transition metal and polycyclic aromatic hydrocarbon contents. Environ Sci Technol.

[41] Ghio AJ, Stonehuerner J, Dailey LA, Carter JD (1999). Metals associated with both the water-soluble and insoluble fractions of an ambient air pollution particle catalyze an oxidative stress. Inhal Toxicol.

[42] Gliga AR, Skoglund S, Wallinder IO, Fadeel B, Karlsson HL (2014). Size-dependent cytotoxicity of silver nanoparticles in human lung cells: the role of cellular uptake, agglomeration and Ag release. Part Fibre Toxicol.

[43] Dye JA, Lehmann JR, McGee JK, Winsett DW, Ledbetter AD, Everitt JI, Ghio AJ, Costa DL (2001). Acute pulmonary toxicity of particulate matter filter extracts in rats: coherence with epidemiologic studies in Utah Valley residents. Environ Health Perspect.

[44] O’Neal SL, Zheng W (2015). Manganese toxicity upon overexposure: a decade in review. Curr Environ Health Rep.

[45] Zhao P, Zhong W, Ying X, Yuan Z, Fu J, Zhou Z (2008). Manganese chloride-induced G0/G1 and S phase arrest in A549 cells. Toxicology.

[46] Kim YH, Fazlollahi F, Kennedy IM, Yacobi NR, Hamm-Alvarez SF, Borok Z, Kim KJ, Crandall ED (2010). Alveolar epithelial cell injury due to zinc oxide nanoparticle exposure. Am J Respir Crit Care Med.

[47] Riley MR, Boesewetter DE, Turner RA, Kim AM, Collier JM, Hamilton A (2005). Comparison of the sensitivity of three lung derived cell lines to metals from combustion derived particulate matter. Toxicol In Vitro.

[48] Becker S, Dailey LA, Soukup JM, Grambow SC, Devlin RB, Huang YC (2005). Seasonal variations in air pollution particle-induced inflammatory mediator release and oxidative stress. Environ Health Perspect.

[49] Hetland RB, Cassee FR, Lag M, Refsnes M, Dybing E, Schwarze PE (2005). Cytokine release from alveolar macrophages exposed to ambient particulate matter: heterogeneity in relation to size, city and season. Part Fibre Toxicol.

