# Supplementary figures and images for: Contrasting biological potency of particulate matter collected at sites impacted by distinct industrial sources
Source: Part Fibre Toxicol. 2016 Dec 1;13:65. doi: 10.1186/s12989-016-0176-y (PMC5134226; doi:10.1186/s12989-016-0176-y)

# A) Water Soluble Metals

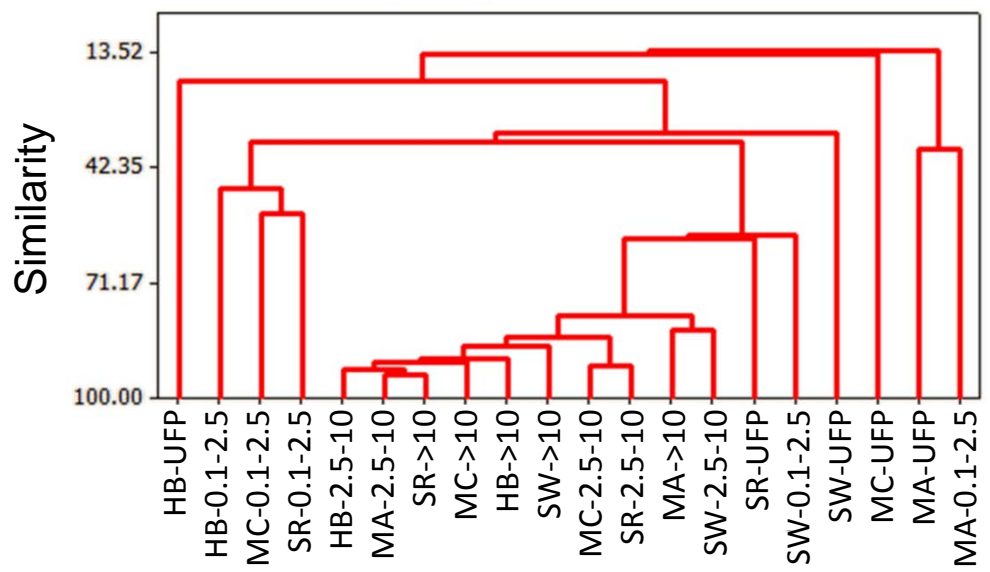

# B) Total Metals

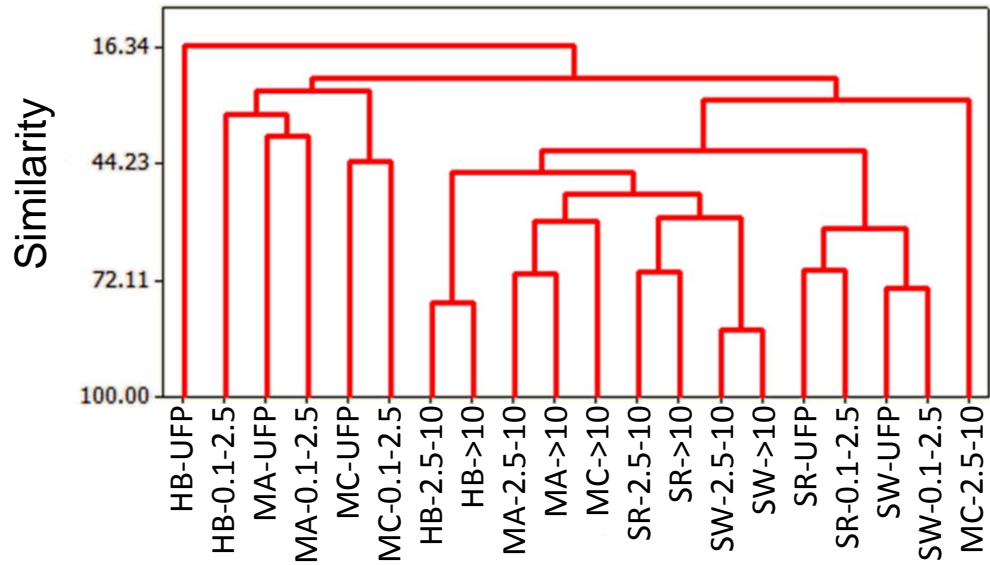

# C) Non-water Soluble

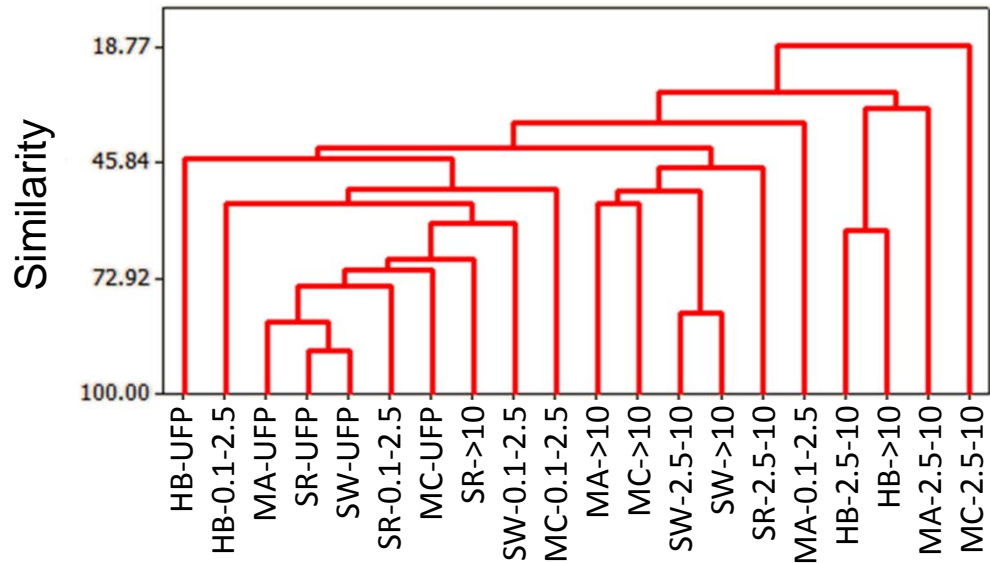

Supplement: Additional file 3: — Clustering of particulate matter samples according to metal content. Size-fractionated particles collected in the vicinity of industrial sites were clustered according to A) water-soluble metals, B) total metals, and C) non-water-soluble (NWS) metals (average linkage, Pearson correlation coefficient distance). (PDF 200 kb) [file 12989_2016_176_MOESM3_ESM.pdf]

### A) Water Soluble Elements

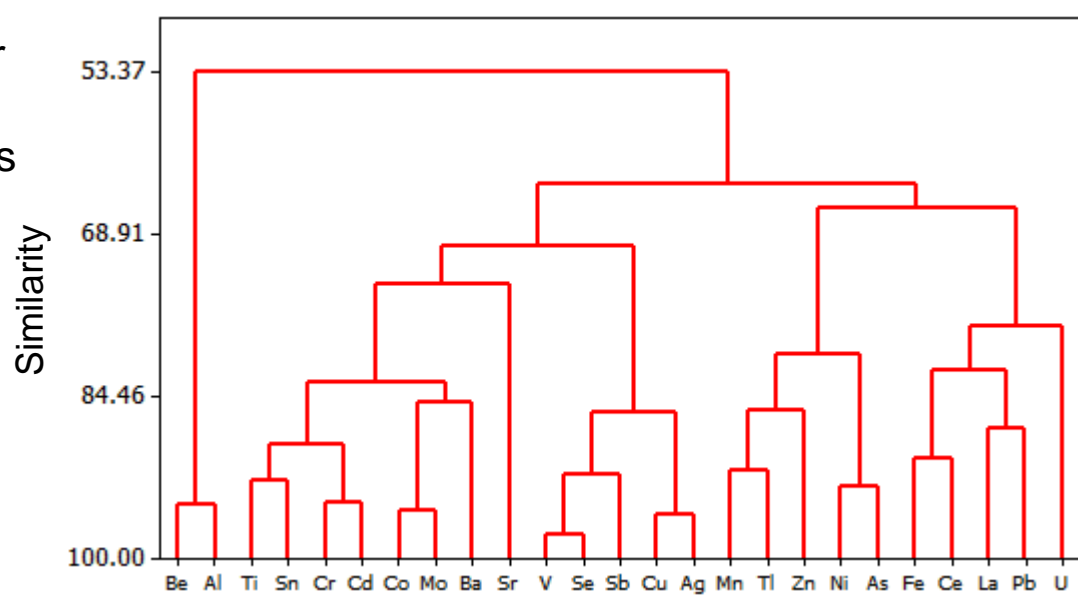

### B) Non-water Soluble Elements

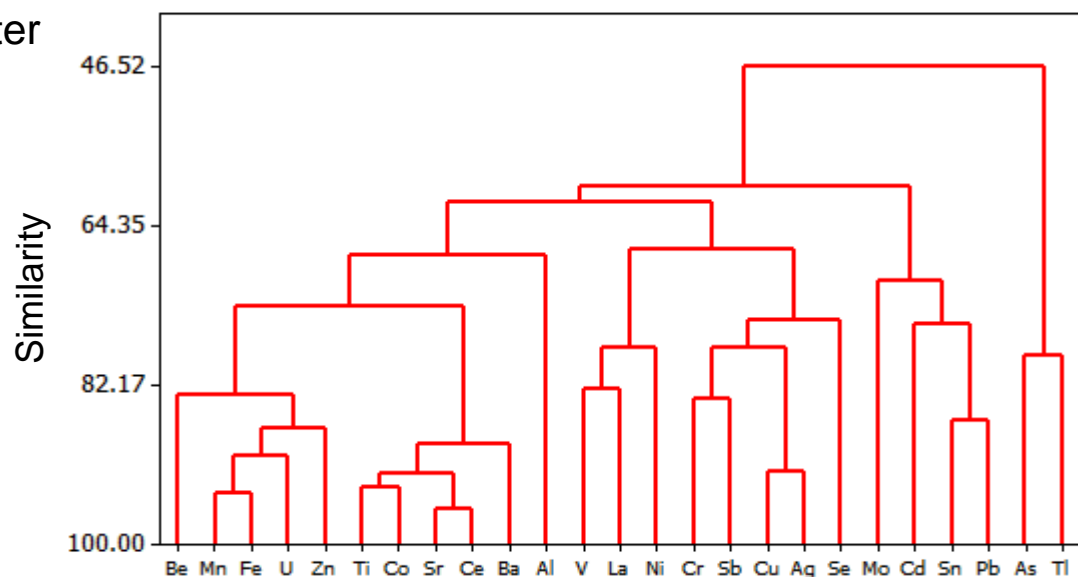

### C) Total Elements

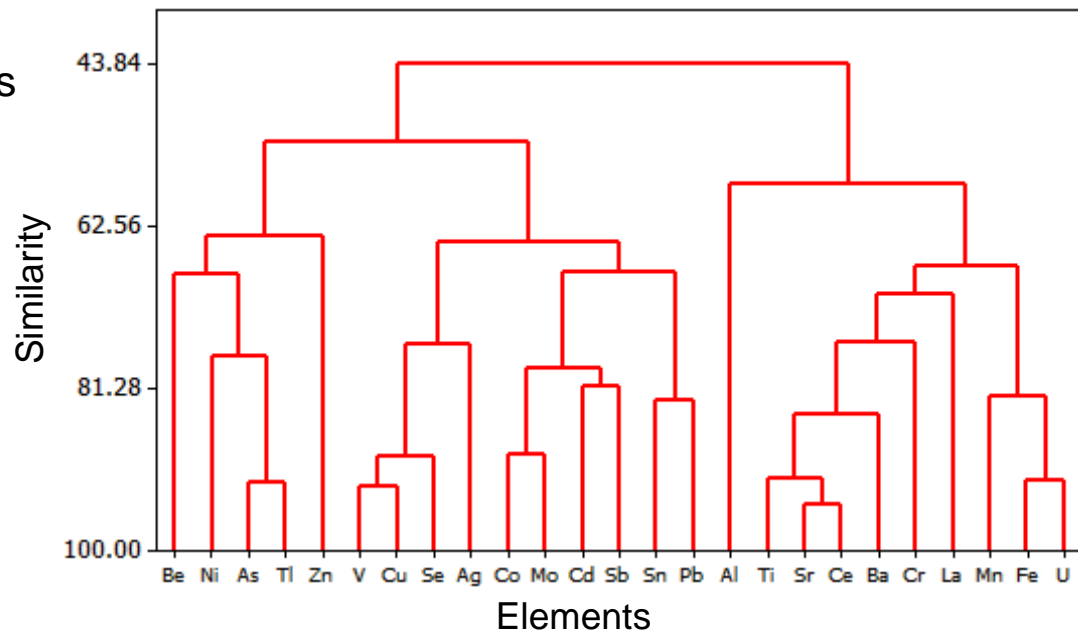

Supplement: Additional file 4: — Clustering of elements according to water soluble, non-water- soluble and total elements. A) Water-soluble (WS), B) non-water soluble (NWS) and C) total metals were clustered to reveal the associations (covariance) among sets of elements. (PDF 22 kb) [file 12989_2016_176_MOESM4_ESM.pdf]

# J774 LDH Release

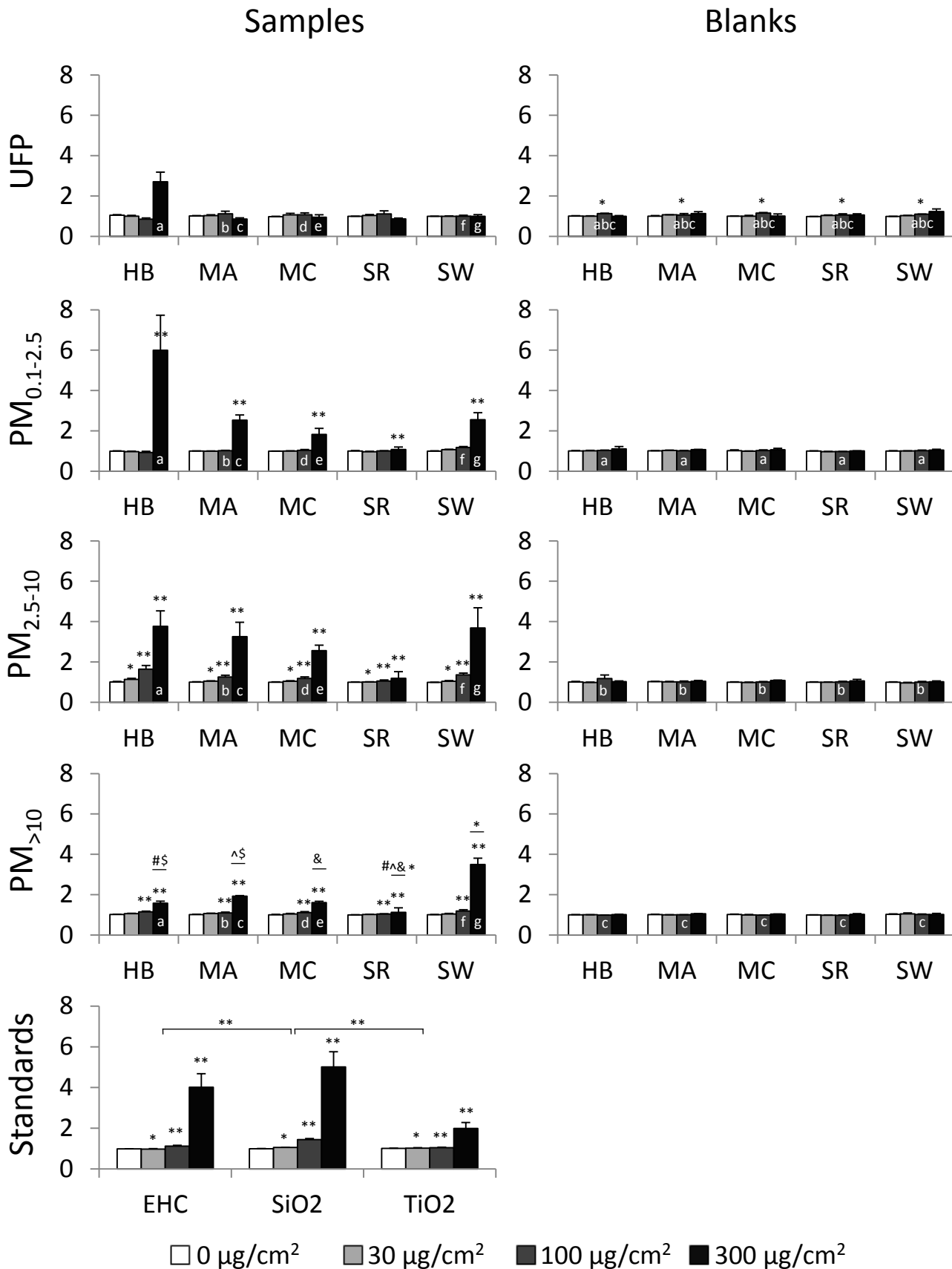

Supplement: Additional file 6: — Cytotoxic responses of J774A.1 cells to 24 h exposure to size-fractionated particulate matter collected in the vicinity of industrial sites according to the LDH release assay. Lactate dehydrogenase (LDH) release into cell culture supernatants of J774A.1 cells exposed for 24 h to size-fractionated and standard reference particles (left-side) and extracts from corresponding field blank filters that were transported to each site but remained unexposed to ambient air (right side). Data represent LDH release adjusted for total cellular LDH content. Values are presented as average fold-effect (FE) over control ± standard error (n = 3 independent experiments). HB, Hamilton Beach steel mill; MA, Montréal AIEM petrochemical refinery; MC, Montréal copper smelter; SR, Sarnia petrochemical refinery; SW, Shawinigan aluminum smelter. Size-fractionated and standard reference particles. Three-way ANOVA (Size-fractionated particles): Site x Dose (p < 0.001) and Size x Dose (p < 0.001) interactions. Asterisks represent significant pairwise comparisons (Holm-Sidak) as follows: doses within Size significantly different from 0 μg/cm2 (*p < 0.05, **p < 0.001). Letters (a-g) represent doses within Site significantly different from 0 μg/cm2 (p < 0.05). Bars with the same symbol (#,@,&,^,$) indicate sites within Dose that are significantly different from one another (p < 0.05). Two-way ANOVA (Standards): Particle (p < 0.001) and Dose (p < 0.001) main effects. Asterisks represent significant pairwise comparisons (Holm-Sidak) as follows: doses significantly different from 0 μg/cm2, or particles significantly different from one another as indicated by brackets (*p < 0.05, **p < 0.001). Field blanks. Three way ANOVA: Size x Dose (p = 0.021) interaction. Asterisks represent doses within Size significantly different from 0 μg/cm2 (*p < 0.05). Letters (a-c) represent sizes within Dose significantly different from one another (p < 0.05). (PDF 55 kb) [file 12989_2016_176_MOESM6_ESM.pdf]

# A549 Resazurin Reduction

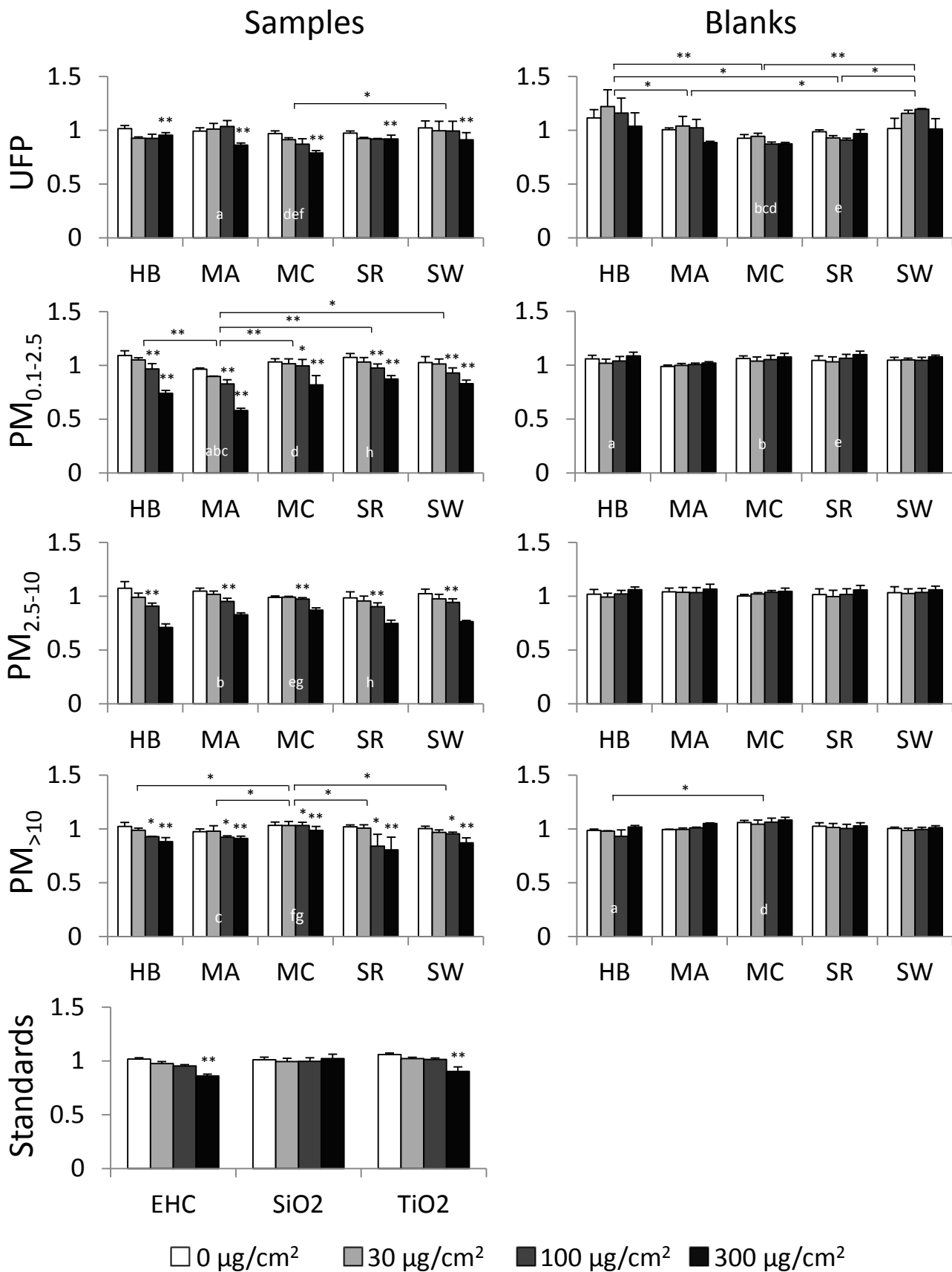

Supplement: Additional file 7: — Cytotoxic responses of A549 cells to 24 h exposure to size-fractionated particulate matter collected in the vicinity of industrial sites according to the resazurin reduction assay. Metabolic reduction of non-fluorescent resazurin in A549 cells exposed for 24 h to size-fractionated and standard reference particles (left-side) and extracts from corresponding field blank filters that were transported to each site but remained unexposed to ambient air (right-side). Values are presented as average fold-effect (FE) over control ± standard error (n = 3 independent experiments). HB, Hamilton Beach steel mill; MA, Montréal AIEM petrochemical refinery; MC, Montréal copper smelter; SR, Sarnia petrochemical refinery; SW, Shawinigan aluminum smelter. Size-fractionated and standard reference particles. Three-way ANOVA (Size-fractionated particles): Site x Size (p < 0.001) and Size x Dose (p = 0.019) interactions. Asterisks represent significant pairwise comparisons (Holm-Sidak) as follows: doses within Site significantly different from 0 μg/cm2, or sites within Size significantly different from one another as indicated by brackets (*p < 0.05, **p < 0.001). Letters (a-h) represent sizes within Site significantly different from one another (p < 0.05). Two-way ANOVA (Standards): Particle x Dose interaction (p = 0.012). Asterisks represent significant pairwise comparisons (Holm-Sidak) as follows: doses within Particles significantly different from 0 μg/cm2 (**p < 0.001). Letters (a,b) represent sites within Dose significantly different from one another (p < 0.5). Field blanks. Three-way ANOVA: Site x Size (p < 0.001) interaction. Asterisks represent significant pairwise comparisons (Holm-Sidak) as follows: sites within Size significantly different from one another as indicated by brackets (*p < 0.05, **p < 0.001). Letters (a-e) represent sizes within Site significantly different from one another (p < 0.05). (PDF 54 kb) [file 12989_2016_176_MOESM7_ESM.pdf]

# A549 LDH Release

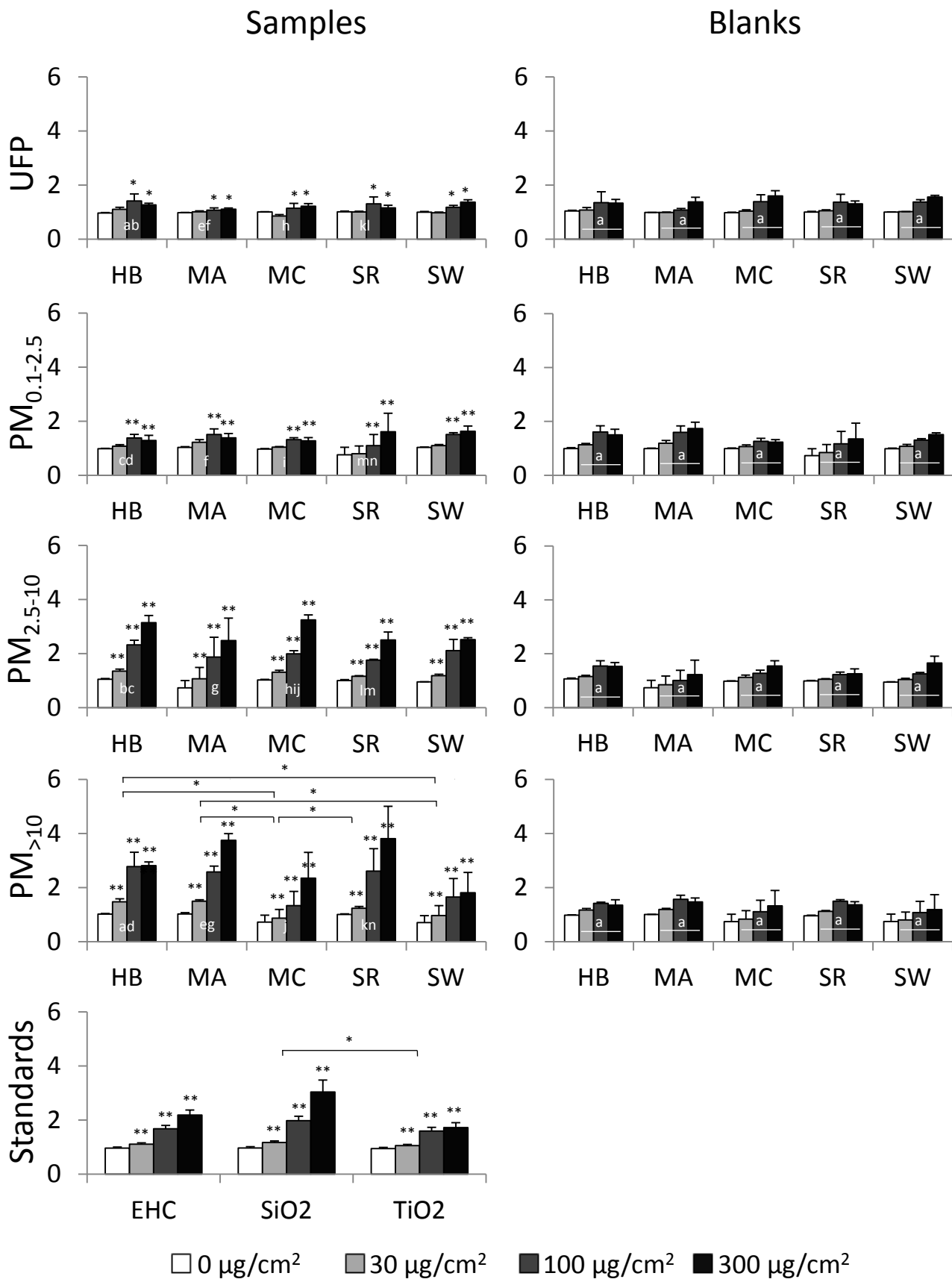

Supplement: Additional file 8: — Cytotoxic responses of A549 cells to 24 h exposure to size-fractionated particulate matter collected in the vicinity of industrial sites according to the LDH release assay. Lactate dehydrogenase (LDH) release into cell culture supernatants of A549 cells exposed for 24 h to size-fractionated and standard reference particles (left-side) and extracts from corresponding field blank filters that were transported to each site but remained unexposed to ambient air (right side). Data represent LDH release, adjusted for total cellular LDH content. Values are presented as average fold-effect (FE) over control ± standard error (n = 3). HB, Hamilton Beach steel mill; MA, Montréal AIEM petrochemical refinery; MC, Montréal copper smelter; SR, Sarnia petrochemical refinery; SW, Shawinigan aluminum smelter. Size-fractionated and standard reference particles. Three-way ANOVA (Size-fractionated particles): Site x Size (p = 0.002) and Size x Dose (p = 0.018) interactions. Asterisks represent significant pairwise comparisons (Holm-Sidak) as follows: doses within Size significantly different from 0 μg/cm2, or sites within Size significantly different from one another as indicated by brackets (*p < 0.05, **p < 0.001). Letters (a-n) represent sizes within Site significantly different from one another (p < 0.05). Two-way ANOVA (Standards): Particle (p = 0.002) and Dose (p < 0.001) main effects. Asterisks represent significant pairwise comparisons (Holm-Sidak) as follows: doses significantly different from 0 μg/cm2, or particles significantly different from one another as indicated by brackets (*p < 0.05, **p < 0.001). Field blanks. Three-way ANOVA: Dose (p < 0.001) main effect. Letter (a) spanned by a line represents doses significantly different from 0 μg/cm2 (p < 0.001). (PDF 55 kb) [file 12989_2016_176_MOESM8_ESM.pdf]

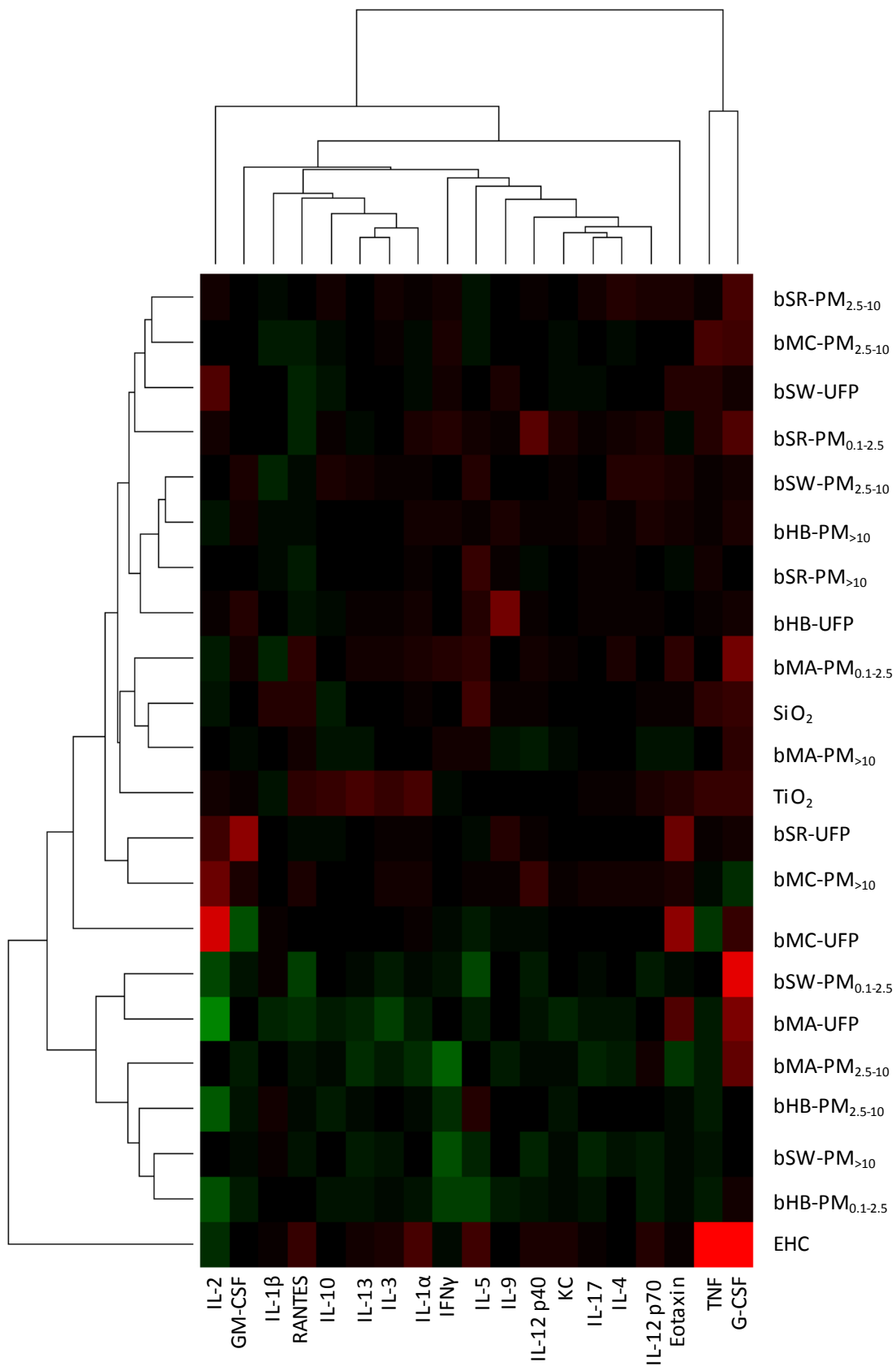

Supplement: Additional file 9: — Inflammatory response to field blank filter extracts. Hierarchical clustering of particles according to cytokine response in J774A.1 cells exposed to field blank filter extracts. The heat map displays particle potency estimates determined for each cytokine from the slope of the dose-effect relationship. Red, increased expression; green, decreased expression. bHB, field blank from Hamilton Beach steel mill; bMA, field blank from Montréal petrochemical refinery; MC, field blank from Montréal copper smelter; SR, field blank from Sarnia petrochemical refinery; SW, field blank from Shawinigan aluminum smelter. (PDF 84 kb) [file 12989_2016_176_MOESM9_ESM.pdf]

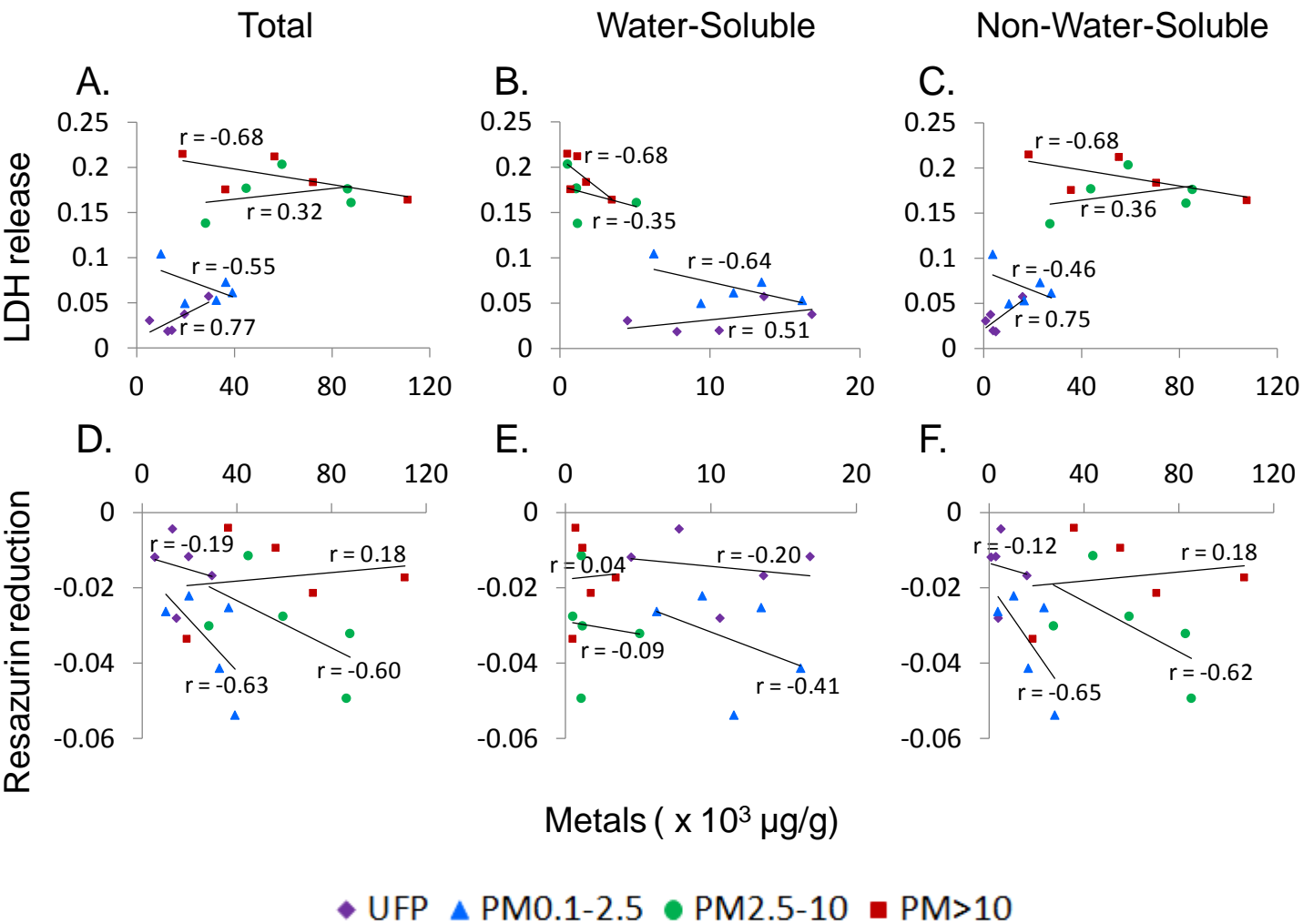

Supplement: Additional file 10: — Association of biological effects in A549 cells with metal content in size-fractionated particles. Cytotoxic potencies according to lactate dehydrogenase (LDH) release and resazurin reduction were regressed against total, water-soluble, and non-water-soluble metals. Pearson product–moment correlation coefficient r-values are presented. LDH release. A) Total metals. UFP, r = 0.77, p = 0.13; PM0.1–2.5, r = −0.55, p = 0.34; PM2.5–10, r = 0.32, p = 0.60; PM>10, r = −0.68, p = 0.21. B) Water-soluble metals. UFP, r = 0.51, p = 0.38; PM0.1–2.5, r = −0.64, p = 0.25; PM2.5–10, r = −0.35, p = 0.57; PM>10, r = −0.68, p = 0.20. C) Non-water-soluble metals. UFP, r = 0.75, p = 0.14; PM0.1–2.5, r = −0.46, p = 0.43; PM2.5–10, r = 0.36, p = 0.55; PM>10, r = −0.68, p = 0.21. Resazurin reduction. D) UFP, r = −0.19, p = 0.76; PM0.1–2.5, r = −0.63, p = 0.26; PM2.5–10, r = −0.60, p = 0.28; PM>10,r = 0.18, p = 0.78. Water-soluble metals. UFP, r = −0.20, p = 0.74; PM0.1–2.5, r = −0.41, p = 0.49; PM2.5–10, r = −0.09, p = 0.88; PM>10, r = 0.04, p = 0.95. Non-water-soluble metals. UFP, r = −0.12, p = 0.84; PM0.1–2.5, r = −0.65, p = 0.24; PM2.5–10, r = −0.62, p = 0.26; PM>10, r = 0.18, p = 0.77. (PDF 43 kb) [file 12989_2016_176_MOESM10_ESM.pdf]
