# Supplementary material for: Contrasting biological potency of particulate matter collected at sites impacted by distinct industrial sources
Source: Part Fibre Toxicol. 2016 Dec 1;13:65. doi: 10.1186/s12989-016-0176-y (PMC5134226; doi:10.1186/s12989-016-0176-y)
Supplement: Additional file 5: — Cytotoxic responses of J774A.1 cells to 24 h exposure to size-fractionated particulate matter collected in the vicinity of industrial sites according to the resazurin reduction assay. Metabolic reduction of non-fluorescent resazurin in J774A.1 cells exposed for 24 h to size-fractionated and standard reference particles (left side) and extracts from corresponding field blank filters that were transported to each site but remained unexposed to ambient air (right side). Values are presented as average fold-effect (FE) over control ± standard error (n = 3 independent experiments). HB, Hamilton Beach steel mill; MA, Montréal petrochemical refinery; MC, Montréal copper smelter; SR, Sarnia petrochemical refinery; SW, Shawinigan aluminum smelter. Size-fractionated and standard reference particles. Three-way ANOVA (Size-fractionated particles): Site x Dose (p = 0.002) and Size x Dose (p < 0.001) interactions. Asterisks represent significant pairwise comparisons (Holm-Sidak) as follows: doses within Site significantly different from 0 μg/cm2, or sites within Size significantly different from one another as indicated by brackets (*p < 0.05, **p < 0.001). Letters (a-e) represent sizes within Site significantly different from one another (p < 0.05). Two-way ANOVA (Standards): Particle (p < 0.001) and Dose (p < 0.001) main effects. Asterisks represent significant pairwise comparisons (Holm-Sidak) as follows: doses significantly different from 0 μg/cm2, or particles significantly different from one another as indicated by brackets (*p < 0.05, **p < 0.001). Field blanks. Three-way ANOVA: Site x Size (p = 0.05) interaction. No significant pairwise comparisons (Holm-Sidak) were observed. (PDF 52 kb) [file 12989_2016_176_MOESM5_ESM.pdf]

J774 Resazurin Reduction

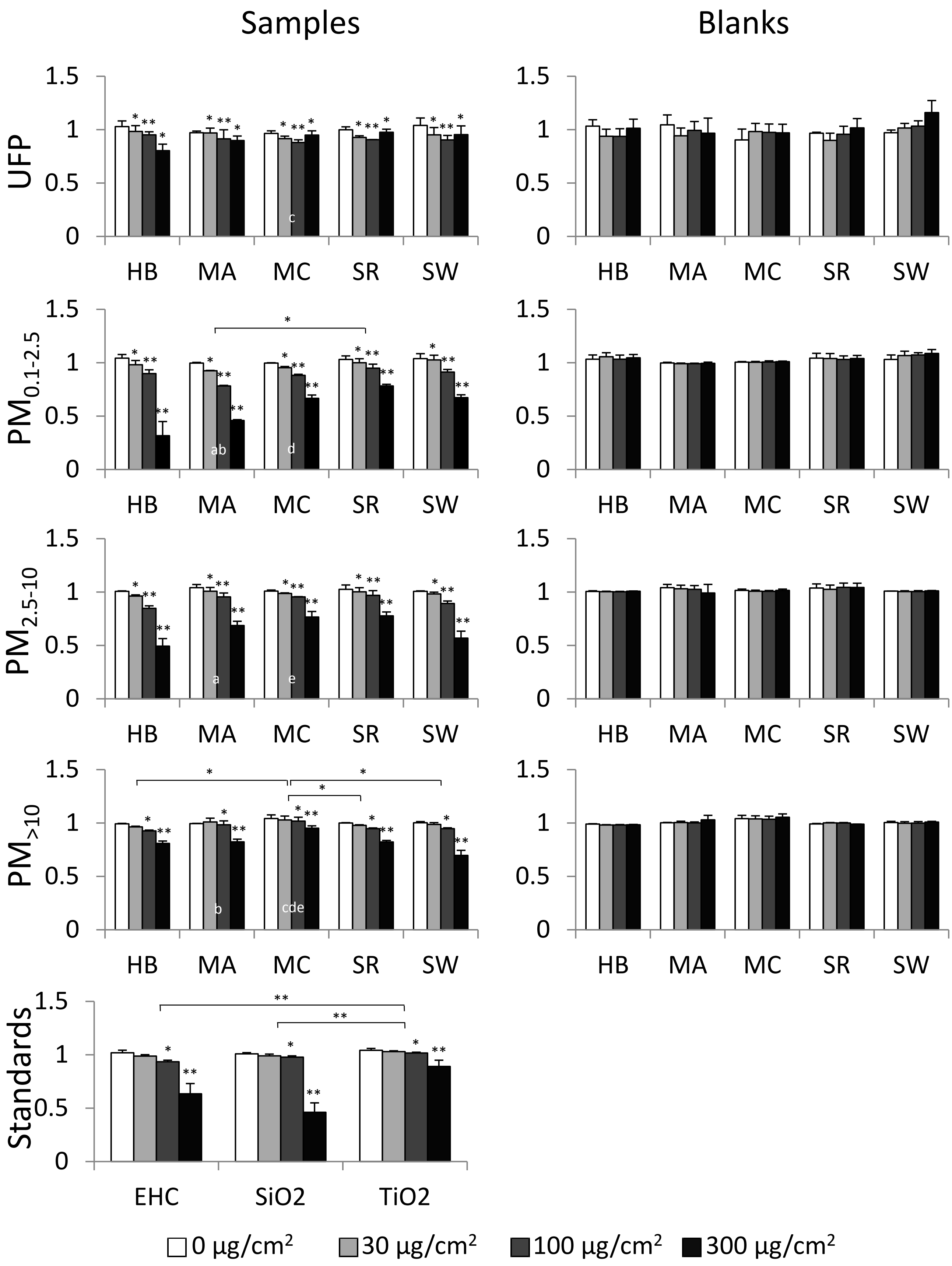

Thomson et. al. Additional File 5
