# Supplementary material for: Contrasting biological potency of particulate matter collected at sites impacted by distinct industrial sources
Source: Part Fibre Toxicol. 2016 Dec 1;13:65. doi: 10.1186/s12989-016-0176-y (PMC5134226; doi:10.1186/s12989-016-0176-y)
Supplement: Additional file 11: — Regressions of biological potency against total metals, water-soluble metals, non-water-soluble metals, endotoxin, and PAHs. Table of Pearson correlations and p-values. (PDF 71 kb) [file 12989_2016_176_MOESM11_ESM.pdf]

	Total PAHs					Volatile PAHs					Semi-volatile PAHs					Particle-associated PAHs					Endotoxin				
	J774 cells			A549 cells		J774 cells			A549 cells		J774 cells			A549 cells		J774 cells			A549 cells		J774 cells			A549 cells	
Size	AB	LDH	Inflam. Pot.	AB	LDH	AB	LDH	Inflam. Pot.	AB	LDH	AB	LDH	Inflam. Pot.	AB	LDH	AB	LDH	Inflam. Pot.	AB	LDH	AB	LDH	Inflam. Pot.	AB	LDH
UFP	N/A	N/A	N/A	N/A	N/A	N/A	N/A	N/A	N/A	N/A	N/A	N/A	N/A	N/A	N/A	N/A	N/A	N/A	N/A	N/A	-0.26	0.39	0.42	0.44	0.17
$PM_{0.1-2.5}$	-0.29	0.74	-0.07	0.26	-0.53	0.31	0.05	0.86**	0.38	0.16	-0.3	0.63	-0.5	0.23	-0.53	-0.53	0.81**	-0.41	-0.01	-0.62	-0.44	0.44	0.33	-0.22	**-0.90***
$PM_{2.5-10}$	-0.38	0.47	-0.07	-0.11	-0.17	0.22	0.01	0.56	0.21	0.72	-0.38	0.42	-0.13	-0.14	-0.24	-0.39	0.42	-0.14	-0.14	-0.27	-0.63	0.69	**0.94***	-0.47	0.79
$PM_{>10}$	-0.7	0.81**	-0.42	-0.04	-0.6	0.55	-0.71	-0.73	-0.25	0.57	-0.82**	**0.92***	-0.13	-0.01	-0.66	-0.55	0.85**	-0.38	0.21	-0.76	-0.65	0.44	0.64	-0.28	0.15
All	-0.19	**0.52***	0.01	-0.0001	0.03	0.4	-0.2	0.37	0.2	0.5**	-0.17	0.46**	0.02	0.01	0.01	-0.5**	**0.63***	-0.29	-0.19	-0.33	-0.33	**0.56***	**0.85***	-0.29	**0.71***

***p < 0.05**

**p<0.1
